# Real-world Cardiovascular Outcomes Associated With Degarelix vs Leuprolide for Prostate Cancer Treatment

**DOI:** 10.1001/jamanetworkopen.2021.30587

**Published:** 2021-10-22

**Authors:** Joshua D. Wallach, Yihong Deng, Rozalina G. McCoy, Sanket S. Dhruva, Jeph Herrin, Alyssa Berkowitz, Eric C. Polley, Kenneth Quinto, Charu Gandotra, William Crown, Peter Noseworthy, Xiaoxi Yao, Nilay D. Shah, Joseph S. Ross, Timothy D. Lyon

**Affiliations:** 1 Department of Environmental Health Sciences, Yale School of Public Health, New Haven, Connecticut; 2 Robert D. and Patricia E. Kern Center for the Science of Health Care Delivery, Mayo Clinic, Rochester, Minnesota; 3 Division of Community Internal Medicine, Department of Medicine, Mayo Clinic, Rochester, Minnesota; 4 Division of Health Care Policy & Research, Department of Health Sciences Research, Mayo Clinic, Rochester, Minnesota; 5 Section of Cardiology, San Francisco Veterans Affairs Health Care System, San Francisco, California; 6 Department of Medicine, UCSF School of Medicine, San Francisco, California; 7 Section of Cardiovascular Medicine, Yale School of Medicine, New Haven, Connecticut; 8 Flying Buttress Associates, Charlottesville, Virginia; 9 Center for Outcomes Research and Evaluation, Yale–New Haven Health, New Haven, Connecticut; 10 Division of Biomedical Statistics and Informatics, Department of Health Sciences Research, Mayo Clinic, Rochester, Minnesota; 11 Office of Medical Policy, Center for Drug Evaluation and Research, US Food and Drug Administration, Silver Springs, Maryland; 12 Office of New Drugs, Center for Drug Evaluation and Research, US Food and Drug Administration, Silver Springs, Maryland; 13 Florence Heller Graduate School, Brandeis University, Waltham, Massachusetts; 14 Department of Cardiovascular Medicine, Mayo Clinic, Rochester, Minnesota; 15 Department of Internal Medicine, Yale School of Medicine, New Haven, Connecticut; 16 Department of Health Policy and Management, Yale School of Public Health, New Haven, Connecticut; 17 Department of Urology, Mayo Clinic, Jacksonville, Florida

## Abstract

**Importance:**

With a growing interest in the use of real-world evidence for regulatory decision-making, it is important to understand whether real-world data can be used to emulate the results of randomized clinical trials.

**Objective:**

To use electronic health record and administrative claims data to emulate the ongoing PRONOUNCE trial (A Trial Comparing Cardiovascular Safety of Degarelix Versus Leuprolide in Patients With Advanced Prostate Cancer and Cardiovascular Disease).

**Design, Setting, and Participants:**

This retrospective, propensity-matched cohort study included adult men with a diagnosis of prostate cancer and cardiovascular disease who initiated either degarelix or leuprolide between December 24, 2008, and June 30, 2019. Participants were commercially insured individuals and Medicare Advantage beneficiaries included in a large US administrative claims database.

**Exposures:**

Degarelix or leuprolide.

**Main Outcomes and Measures:**

The primary end point was time to first occurrence of a major adverse cardiovascular event (MACE), defined as death due to any cause, myocardial infarction, or stroke, analogous to the PRONOUNCE trial. Secondary end points were time to death due to any cause, myocardial infarction, stroke, and angina. Cox proportional hazards regression was used to evaluate primary and secondary end points.

**Results:**

A total of 32 172 men initiated degarelix or leuprolide for prostate cancer; of them, 9490 (29.5%) had cardiovascular disease, and 7800 (24.2%) met the PRONOUNCE trial eligibility criteria and were included in this study. Overall, 165 participants (2.1%) were Asian, 1390 (17.8%) were Black, 663 (8.5%) were Hispanic, and 5258 (67.4%) were White. The mean (SD) age was 74.4 (7.4) years. Among 2226 propensity score–matched patients, no significant difference was observed in the risk of MACE for patients taking degarelix vs those taking leuprolide (10.18 vs 8.60 events per 100 person-years; hazard ratio [HR], 1.18; 95% CI, 0.86-1.61). Degarelix was associated with a higher risk of death from any cause (HR, 1.48; 95% CI, 1.01-2.18) but not of myocardial infarction (HR, 1.16; 95% CI, 0.60-2.25), stroke (HR, 0.92; 95% CI, 0.45-1.85), or angina (HR, 1.36; 95% CI, 0.43-4.27).

**Conclusions and Relevance:**

In this emulation of a clinical trial of men with cardiovascular disease undergoing treatment for prostate cancer, degarelix was not associated with a lower risk of cardiovascular events than leuprolide. Comparison of these data with PRONOUNCE trial results, when published, will help enhance our understanding of the appropriate role of using real-world data to emulate clinical trials.

## Key Points

**Question:**

Can real-world data regarding the use of degarelix and leuprolide be used to emulate the forthcoming PRONOUNCE trial, a phase 3b trial comparing the cardiovascular safety of degarelix vs leuprolide among patients with prostate cancer and cardiovascular disease?

**Findings:**

In this cohort study of 2226 propensity score–matched men with prostate cancer taking degarelix or leuprolide, no significant difference was observed in the risk of a major adverse cardiovascular event.

**Meaning:**

These findings suggest that real-world data are increasingly available and useful for medical product evaluation, including for emulating clinical trials to understand products’ use in clinical practice and the associated benefits and harms of treatment.

## Teaser

This cohort study uses electronic health record and administrative claims data to emulate the ongoing PRONOUNCE trial, which compares the cardiovascular safety of degarelix and leuprolide in men with cardiovascular disease and advanced prostate cancer.

## Introduction

Prostate cancer is the most common solid organ malignant neoplasm and the second leading cause of cancer death among men in the United States.^1^ Androgen deprivation therapy (ADT) is a cornerstone of treatment for many men with prostate cancer^2,3,4^; however, there is evidence that ADT may increase the risk of cardiovascular morbidity and mortality, especially among patients with preexisting cardiovascular disease.^5,6^ Secondary analyses of multiple randomized clinical trials (RCTs) have suggested that patients treated with degarelix, a gonadotropin-releasing hormone (GnRH) antagonist, may have a lower risk of cardiovascular events than those treated with GnRH agonists, such as leuprolide. Trials have also suggested that relugolix, another GnRH antagonist, is associated with a lower risk of major adverse cardiovascular events (MACEs) compared with leuprolide.^7^ However, no prior trials directly comparing degarelix and leuprolide have been published with cardiovascular events as a primary end point.^8,9,10^ As a result, the long-term relationship between ADT and cardiovascular risk, as well as the potential differences between degarelix and leuprolide, remains uncertain.^11,12^ To address this question, a Phase 3b RCT comparing the cardiovascular safety of degarelix and leuprolide among patients with prostate cancer and cardiovascular disease is ongoing (the PRONOUNCE trial [A Trial Comparing Cardiovascular Safety of Degarelix Versus Leuprolide in Patients With Advanced Prostate Cancer and Cardiovascular Disease]).^13^

Real-world data (RWD) are increasingly available to support medical product evaluations, including for regulatory decision-making, to better understand use and associated benefits and risks of treatment. While double-masked RCTs, like PRONOUNCE, are the gold standard to evaluate the efficacy and safety of medical products, they face operational challenges and have important limitations that undermine their generalizability to real-world clinical practice. For instance, RCTs are often expensive, face recruitment and retention difficulties, and take a long time to complete.^14^ Furthermore, RCTs often have strict inclusion and exclusion criteria, which can affect the number of participants from racial and ethnic minority groups, who are known to be underrepresented in prostate cancer trials and clinical trials in general.^15,16^ While PRONOUNCE planned to enroll 900 patients when it started in 2016, only 545 patients will have been recruited by estimated study completion in 2021,^13^ which may limit the trial’s ability to answer the safety questions of interest.

The noted shortcomings of RCTs have increased interest in rigorously designed observational studies using RWD.^16,17^ While RWD are useful for describing how medications are actually used in clinical practice, including patient demographic characteristics, comorbidities, and concurrent treatments,^18,19^ it remains unclear whether RWD and observational methods can be used to address the same clinical questions evaluated by RCTs and potentially support regulatory decision-making.^16,18^ Many studies have replicated the results of completed RCTs using RWD and observational methods^20,21,22,23,24,25,26^; however, such an approach is potentially prone to design, selection, and/or measurement biases, as these analyses are planned after the trials’ results are already known. The ability of routinely collected electronic health record (EHR) or administrative claims data to anticipate the end points of ongoing clinical trials, including emulating the characteristics of enrolled patients and the results of the primary research questions, has not been well examined.

In this study, we sought to evaluate whether a large cohort of US patients with commercial insurance could be used to assess the proportion and characteristics of patients in routine practice who met the PRONOUNCE trial inclusion criteria. Furthermore, we applied observational research methods to emulate the PRONOUNCE trial’s anticipated results. This study followed the target trial framework and was conceived and conducted prior to the release of the PRONOUNCE trial results.^27^

## Methods

The Mayo Clinic institutional review board exempted this study from review and the requirement for informed consent because it used preexisting, deidentified data. This study was conducted and reported according to the Reporting of Studies Conducted Using Observational Routinely Collected Data (RECORD) and International Society for Pharmacoeconomics and Outcomes Research (ISPOR) reporting guidelines.^28,29^ The protocol is available online.^30^

### Study Design and Population

This retrospective cohort study used deidentified administrative claims data from OptumLabs Data Warehouse (OLDW), which includes EHR, medical and pharmacy claims, laboratory results, and enrollment records for commercial and Medicare Advantage enrollees in the United States. OLDW contains longitudinal health information on enrollees, representing a diverse mixture of ages, racial and ethnic groups, and geographical regions across the United States.^31^ Inclusion and exclusion criteria for the PRONOUNCE trial were identified from ClinicalTrials.gov and the trial protocol and were adopted and applied to beneficiaries included in OLDW (eAppendix in the Supplement).^13^ The cohort included men aged 18 years or older with valid demographic (age and race and ethnicity) data, a history of cardiovascular disease, and a current diagnosis of prostate cancer who initiated degarelix or leuprolide between December 24, 2008 (the Food and Drug Administration [FDA] approval date of degarelix), and June 30, 2019. The date of an individual’s first treatment with degarelix or leuprolide was defined as the index date (ie, new-user design).^32^ Patients were required to have at least 6 months of continuous enrollment with medical and pharmacy coverage before the index date. Patients were followed up until they experienced an end point of interest, reached the maximum anticipated follow-up of the trial (ie, 336 days) or the end of the study period (July 31, 2019), ended insurance coverage (end of patient enrollment), or died. Variables were defined by the presence of a claim with eligible diagnosis codes, procedure codes, and prescription fills, as detailed in the eAppendix in the Supplement.

### End Points

Primary and secondary end points were defined to mirror those of the PRONOUNCE trial. The primary end point was the time from index date to first occurrence of an MACE, a composite end point defined as all-cause death, nonfatal myocardial infarction, or nonfatal stroke (eAppendix in the Supplement).

Secondary end points were time from index date to all-cause death, myocardial infarction, stroke, and unstable angina, as separate end points. In the PRONOUNCE protocol, an additional secondary end point was time to cardiovascular-related death. However, in OLDW, we were unable to distinguish causes of death and stable vs unstable angina; therefore, we evaluated all-cause death and any angina. We used previously published and validated diagnosis and procedure codes to determine myocardial infarction, stroke, and angina (eAppendix in the Supplement). Mortality was identified using the mortality data from OLDW, which is based on the Social Security Death Master File, deceased status from EHR data, death as a reason for disenrollment, and death indicated by inpatient discharge status.

### Other Variables of Interest

Sociodemographic variables, comorbidities, and prior and concurrent medication use during the baseline 6-month period were recorded (Table 1; eAppendix in the Supplement). In the OLDW database, race is derived from ethnicity. Ethnicity is imputed based on a model run by the data supplier using an individual’s name (first, last, middle) and geographic location and then categorized into 5 race values (ie, Asian, Black, Hispanic, unknown, and White). Race and ethnicity were analyzed so that we could include these data in our propensity score (PS) modeling. When available, laboratory results data were queried for pretreatment prostate-specific antigen (PSA) levels and estimated glomerular filtration rate (eGFR), which were used as matching and subgroup variables, respectively. Laboratory results are available for approximately one-third of patients, conditional on the contracts between commercial laboratory providers and the health plan entities whose patients are represented in OLDW.

**Table 1.** Baseline Characteristics Before and After PS Matching

Characteristic	Before PS matching				After PS matching			
	Patients, No. (%)			SMD	Patients, No. (%)			SMD
	Degarelix (n = 1120)	Leuprolide (n = 6680)	Total (N = 7800)		Degarelix (n = 1113)	Leuprolide (n = 1113)	Total (N = 2226)	
Age, y								
Mean (SD)	75.0 (7.6)	74.3 (7.4)	74.4 (7.4)	0.10	75.1 (7.6)	75.0 (7.4)	75.0 (7.5)	0.01
Median (IQR)	75.5 (70.0-81.0)	75.0 (70.0-80.0)	75.0 (70.0-80.0)		76.0 (70.0-81.0)	76.0 (71.0-81.0)	76.0 (70.0-81.0)	
≤54	<11^a^	55 (0.8)	>60^a^	0.04	<11^a^	11 (1.0)	>14^a^	0.05
55-64	113 (10.1)	662 (9.9)	775 (9.9)	0.01	111 (10.0)	96 (8.6)	207 (9.3)	0.05
65-74	>383^a^	2418 (36.2)	>2800^a^	0.03	>380^a^	367 (33.0)	>750^a^	0.03
≥75	613 (54.7)	3545 (53.1)	4158 (53.3)	0.03	611 (54.9)	639 (57.4)	1250 (56.2)	0.05
Race and ethnicity								
Asian	24 (2.1)	141 (2.1)	165 (2.1)	0.00	24 (2.2)	27 (2.4)	51 (2.3)	0.02
Black	209 (18.7)	1181 (17.7)	1390 (17.8)	0.03	207 (18.6)	208 (18.7)	415 (18.6)	0.00
Hispanic	55 (4.9)	608 (9.1)	663 (8.5)	0.17	55 (4.9)	51 (4.6)	106 (4.8)	0.02
White	783 (69.9)	4475 (67.0)	5258 (67.4)	0.06	778 (69.9)	779 (70.0)	1557 (69.9)	0.00
Unknown	49 (4.4)	275 (4.1)	324 (4.2)	0.01	49 (4.4)	48 (4.3)	97 (4.4)	0.00
Geographic region								
Midwest	301 (26.9)	1830 (27.4)	2131 (27.3)	0.01	301 (27.0)	313 (28.1)	614 (27.6)	0.02
Northeast	217 (19.4)	1063 (15.9)	1280 (16.4)	0.09	214 (19.2)	205 (18.4)	419 (18.8)	0.02
South	>495^a^	>3158^a^	>3660	0.05	>493^a^	>489^a^	>993^a^	0.01
West	96 (8.6)	618 (9.3)	714 (9.2)	0.02	94 (8.4)	95 (8.5)	189 (8.5)	0.00
Unknown	<11^a^	<11^a^	<11^a^	0.01	<11^a^	<11^a^	<11^a^	0.00
Serum PSA level^b^								
No. (%)	440 (35.2)	2246 (33.6)	2686 (34.4)	NA	423 (38.0)	423 (38.0)	846 (38.0)	NA
Mean (SD), ng/mL	74.3 (261.2)	48.9 (290.7)	53.0 (286.1)	0.09	70.6 (262.9)	59.3 (341.6)	64.9 (304.7)	0.05
Median (IQR), ng/mL	11.6 (6.2-32.4)	9.2 (5.1-19.8)	9.4 (5.2-21.6)		11.6 (6.2-30.8)	9.9 (5.3-24.4)	11.0 (5.8-27.3)	
eGFR^c^								
No. (%)	433 (34.6)	2353 (35.2)	2786 (35.7)	NA	428 (38.5)	377 (33.9)	805 (36.2)	NA
Mean (SD), mL/min/1.73 m^2^	66.5 (20.5)	66.8 (20.4)	66.8 (20.4)	0.02	66.5 (20.5)	64.9 (21.4)	65.7 (20.9)	0.08
Median (IQR), mL/min/1.73 m^2^	68.3 (52.4-81.9)	68.5 (53.1-82.5)	68.5 (53.0-82.5)		68.1 (52.4-81.9)	67.2 (51.6-80.4)	67.8 (52.1-81.2)	
Prostate biopsy within 6 mos of index date, No. (%)	682 (60.9)	3635 (54.4)	4317 (55.3)	0.13	676 (60.7)	683 (61.4)	1359 (61.1)	0.01
Baseline comorbidities								
Coronary artery disease	593 (52.9)	3424 (51.3)	4017 (51.5)	0.03	593 (53.3)	580 (52.1)	1173 (52.7)	0.02
Chronic kidney disease	167 (14.9)	877 (13.1)	1044 (13.4)	0.05	166 (14.9)	176 (15.8)	342 (15.4)	0.03
Congestive heart failure	191 (17.1)	1041 (15.6)	1232 (15.8)	0.04	190 (17.1)	173 (15.5)	363 (16.3)	0.04
Cerebrovascular disease	159 (14.2)	885 (13.2)	1044 (13.4)	0.03	158 (14.2)	169 (15.2)	327 (14.7)	0.03
Peripheral vascular disease	326 (29.1)	2001 (30.0)	2327 (29.8)	0.02	325 (29.2)	322 (28.9)	647 (29.1)	0.01
Obesity^d^	71 (6.3)	451 (6.8)	522 (6.7)	0.02	70 (6.3)	62 (5.6)	132 (5.9)	0.03
Atrial fibrillation	191 (17.1)	1104 (16.5)	1295 (16.6)	0.01	189 (17.0)	197 (17.7)	386 (17.3)	0.02
Sleep apnea	107 (9.6)	682 (10.2)	789 (10.1)	0.02	105 (9.4)	107 (9.6)	212 (9.5)	0.01
Hypertension	882 (78.8)	5290 (79.2)	6172 (79.1)	0.01	876 (78.7)	867 (77.9)	1743 (78.3)	0.02
MI	119 (10.6)	696 (10.4)	815 (10.4)	0.01	119 (10.7)	118 (10.6)	237 (10.6)	0.00
Stroke	60 (5.4)	375 (5.6)	435 (5.6)	0.01	59 (5.3)	68 (6.1)	127 (5.7)	0.04
PCI	81 (7.2)	494 (7.4)	575 (7.4)	0.01	81 (7.3)	83 (7.5)	164 (7.4)	0.01
CABG	97 (8.7)	634 (9.5)	731 (9.4)	0.03	97 (8.7)	90 (8.1)	187 (8.4)	0.02
PAD	232 (20.7)	1339 (20.0)	1571 (20.1)	0.02	229 (20.6)	228 (20.5)	457 (20.5)	0.00
Dementia	57 (5.1)	271 (4.1)	328 (4.2)	0.05	55 (4.9)	47 (4.2)	102 (4.6)	0.03
COPD	244 (21.8)	1340 (20.1)	1584 (20.3)	0.04	241 (21.7)	234 (21.0)	475 (21.3)	0.02
Peptic ulcer disease	11 (1.0)	73 (1.1)	84 (1.1)	0.01	11 (1.0)	16 (1.4)	27 (1.2)	0.04
Mild liver disease	78 (7.0)	524 (7.8)	602 (7.7)	0.03	78 (7.0)	77 (6.9)	155 (7.0)	0.00
Diabetes without chronic complication	414 (37.0)	2405 (36.0)	2819 (36.1)	0.02	411 (36.9)	375 (33.7)	786 (35.3)	0.07
Diabetes with chronic complication	182 (16.3)	1011 (15.1)	1193 (15.3)	0.03	180 (16.2)	184 (16.5)	364 (16.4)	0.01
Metastatic solid tumor	251 (22.4)	1097 (16.4)	1348 (17.3)	0.15	247 (22.2)	250 (22.5)	497 (22.3)	0.01
Rheumatic disease	21 (1.9)	149 (2.2)	170 (2.2)	0.03	21 (1.9)	28 (2.5)	49 (2.2)	0.04
Charlson comorbidity score								
Mean (SD)	5.5 (3.2)	5.1 (3.0)	5.1 (3.1)	0.14	5.5 (3.2)	5.5 (3.3)	5.5 (3.3)	0.00
Median (IQR)	5.0 (3.0-8.0)	4.0 (3.0-7.0)	4.0 (3.0-7.0)		5.0 (3.0-8.0)	4.0 (3.0-8.0)	5.0 (3.0-8.0)	
Radiotherapy within 6 mos before index date, No. (%)	19 (1.7)	204 (3.1)	223 (2.9)	0.09	19 (1.7)	17 (1.5)	36 (1.6)	0.01
Use of bicalutamide within 6 mos before index date, No. (%)	0	454 (40.8)	454 (40.8)	NA	NA	NA	NA	NA
Other baseline medications within 6 mos before index date								
Statin	717 (64.0)	4264 (63.8)	4981 (63.9)	0.00	714 (64.2)	723 (65.0)	1437 (64.6)	0.02
Nonstatin lipid-lowering medications	170 (15.2)	976 (14.6)	1146 (14.7)	0.02	169 (15.2)	173 (15.5)	342 (15.4)	0.01
ACEi	396 (35.4)	2458 (36.8)	2854 (36.6)	0.03	391 (35.1)	393 (35.3)	784 (35.2)	0.00
ARB	215 (19.2)	1243 (18.6)	1458 (18.7)	0.02	211 (19.0)	218 (19.6)	429 (19.3)	0.02
ACEi and ARB	593 (52.9)	3620 (54.2)	4213 (54.0)	0.03	588 (52.8)	591 (53.1)	1179 (53.0)	0.01
Sacubitril and valsartan	<11^a^	>15^a^	22 (0.3)	0.00	<11^a^	<11^a^	<11^a^	0.02
Warfarin	102 (9.1)	648 (9.7)	750 (9.6)	0.02	102 (9.2)	96 (8.6)	198 (8.9)	0.02
DOAC	70 (6.3)	298 (4.5)	368 (4.7)	0.08	68 (6.1)	70 (6.3)	138 (6.2)	0.01
β-blockers	581 (51.9)	3211 (48.1)	3792 (48.6)	0.08	577 (51.8)	577 (51.8)	1154 (51.8)	0.00
Loop diuretics	174 (15.5)	1042 (15.6)	1216 (15.6)	0.00	173 (15.5)	160 (14.4)	333 (15.0)	0.03
Aldosterone antagonist	46 (4.1)	230 (3.4)	276 (3.5)	0.04	45 (4.0)	52 (4.7)	97 (4.4)	0.03
Digoxin	28 (2.5)	205 (3.1)	233 (3.0)	0.04	28 (2.5)	30 (2.7)	58 (2.6)	0.01
Calcium channel blocker	294 (26.3)	1666 (24.9)	1960 (25.1)	0.03	291 (26.1)	269 (24.2)	560 (25.2)	0.05
Antiplatelet	214 (19.1)	1219 (18.2)	1433 (18.4)	0.02	211 (19.0)	214 (19.2)	425 (19.1)	0.01
No. of hospitalizations								
0	922 (82.3)	5529 (82.8)	6451 (82.7)	0.01	915 (82.2)	894 (80.3)	1809 (81.3)	0.05
1	153 (13.7)	915 (13.7)	1068 (13.7)	0.00	153 (13.7)	176 (15.8)	329 (14.8)	0.06
≥2	45 (4.0)	236 (3.5)	281 (3.6)	0.03	45 (4.0)	43 (3.9)	88 (4.0)	0.01
No. of ED visits								
0	837 (74.7)	5151 (77.1)	5988 (76.8)	0.06	835 (75.0)	834 (74.9)	1669 (75.0)	0.00
1	164 (14.6)	935 (14.0)	1099 (14.1)	0.02	160 (14.4)	155 (13.9)	315 (14.2)	0.01
≥2	119 (10.6)	594 (8.9)	713 (9.1)	0.06	118 (10.6)	124 (11.1)	242 (10.9)	0.02
Year of cohort entry								
2008	0	<11^a^	<11^a^	NA	0	0	0	NA
2009	0	>369^a^	>369^a^	0.34	0	28 (2.5)	28 (1.3)	0.23
2010	29 (2.6)	441 (6.6)	470 (6.0)	0.19	29 (2.6)	48 (4.3)	77 (3.5)	0.09
2011	61 (5.4)	487 (7.3)	548 (7.0)	0.08	61 (5.5)	39 (3.5)	100 (4.5)	0.10
2012	73 (6.5)	482 (7.2)	555 (7.1)	0.03	73 (6.6)	54 (4.9)	127 (5.7)	0.07
2013	89 (7.9)	473 (7.1)	562 (7.2)	0.03	89 (8.0)	74 (6.6)	163 (7.3)	0.05
2014	95 (8.5)	481 (7.2)	576 (7.4)	0.05	95 (8.5)	75 (6.7)	170 (7.6)	0.07
2015	104 (9.3)	579 (8.7)	683 (8.8)	0.02	104 (9.3)	93 (8.4)	197 (8.8)	0.03
2016	150 (13.4)	742 (11.1)	892 (11.4)	0.07	150 (13.5)	134 (12.0)	284 (12.8)	0.04
2017	198 (17.7)	1027 (15.4)	1225 (15.7)	0.06	197 (17.7)	226 (20.3)	423 (19.0)	0.07
2018	225 (20.1)	1156 (17.3)	1381 (17.7)	0.07	222 (19.9)	235 (21.1)	457 (20.5)	0.03
2019	96 (8.6)	432 (6.5)	528 (6.8)	0.08	93 (8.4)	107 (9.6)	200 (9.0)	0.04
State								
Alabama	34 (3.0)	170 (2.5)	204 (2.6)	0.03	34 (3.1)	31 (2.8)	65 (2.9)	0.02
Arizona	21 (1.9)	107 (1.6)	128 (1.6)	0.02	20 (1.8)	21 (1.9)	41 (1.8)	0.01
California	12 (1.1)	118 (1.8)	130 (1.7)	0.06	12 (1.1)	21 (1.9)	33 (1.5)	0.07
Connecticut	24 (2.1)	138 (2.1)	162 (2.1)	0.01	23 (2.1)	24 (2.2)	47 (2.1)	0.01
Florida	164 (14.6)	1056 (15.8)	1220 (15.6)	0.03	164 (14.7)	157 (14.1)	321 (14.4)	0.02
Georgia	54 (4.8)	495 (7.4)	549 (7.0)	0.11	54 (4.9)	97 (8.7)	151 (6.8)	0.15
Illinois	62 (5.5)	318 (4.8)	380 (4.9)	0.04	62 (5.6)	58 (5.2)	120 (5.4)	0.02
Indian	39 (3.5)	177 (2.6)	216 (2.8)	0.05	39 (3.5)	34 (3.1)	73 (3.3)	0.03
Massachusetts	<11^a^	>90^a^	98 (1.3)	0.11	<11^a^	>11^a^	>11^a^	0.10
Minnesota	12 (1.1)	157 (2.4)	169 (2.2)	0.10	12 (1.1)	29 (2.6)	41 (1.8)	0.11
Missouri	50 (4.5)	275 (4.1)	325 (4.2)	0.02	50 (4.5)	44 (4.0)	94 (4.2)	0.03
North Carolina	>118^a^	>365^a^	498 (6.4)	0.20	124 (11.1)	54 (4.9)	178 (8.0)	0.23
New Jersey	75 (6.7)	234 (3.5)	309 (4.0)	0.15	74 (6.6)	45 (4.0)	119 (5.3)	0.12
New York	89 (7.9)	402 (6.0)	491 (6.3)	0.08	89 (8.0)	92 (8.3)	181 (8.1)	0.01
Ohio	33 (2.9)	331 (5.0)	364 (4.7)	0.10	33 (3.0)	47 (4.2)	80 (3.6)	0.07
Rhode Island	16 (1.4)	116 (1.7)	132 (1.7)	0.03	16 (1.4)	17 (1.5)	33 (1.5)	0.01
South Carolina	29 (2.6)	109 (1.6)	138 (1.8)	0.07	28 (2.5)	21 (1.9)	49 (2.2)	0.04
Tennessee	16 (1.4)	133 (2.0)	149 (1.9)	0.04	16 (1.4)	22 (2.0)	38 (1.7)	0.04
Texas	28 (2.5)	533 (8.0)	561 (7.2)	0.25	28 (2.5)	66 (5.9)	94 (4.2)	0.17
Utah	15 (1.3)	143 (2.1)	158 (2.0)	0.06	15 (1.3)	21 (1.9)	36 (1.6)	0.04
Virginia	<11^a^	>82^a^	>90^a^	0.07	<11^a^	>12^a^	>20^a^	0.08
Wisconsin	74 (6.6)	371 (5.6)	445 (5.7)	0.04	74 (6.6)	64 (5.8)	138 (6.2)	0.04
Other	137 (12.2)	744 (11.1)	881 (11.3)	0.03	135 (12.1)	118 (10.6)	253 (11.4)	0.05

Abbreviations: ACEi, angiotensin-converting-enzyme inhibitor; ARB, angiotensin II receptor blocker; CABG, coronary artery bypass grafting; COPD, chronic obstructive pulmonary disease; DOAC, direct-acting oral anticoagulant; ED, emergency department; eGFR, estimated glomerular filtration rate; IQR, interquartile range; MI, myocardial infarction; NA, not applicable; PAD, peripheral artery disease; PCI, percutaneous coronary intervention; PS, propensity score; PSA prostate-specific antigen; SMD, standardized mean difference.

SI conversion: To convert PSA level to micrograms per liter, multiply by 1.0.

^a^ Cell suppression based on OptumLabs cell size suppression rules. Categories with fewer than 11 individuals are masked to protect patient confidentiality.

^b^ Patients with PSA and without PSA levels were matched separately.

^c^ eGFR was used for the subgroup analyses and not for matching purposes.

^d^ Obesity defined as a body mass index (calculated as weight in kilograms divided by height in meters squared) of 30 or greater.

### Statistical Analysis

#### Main Analysis

The full statistical analysis plan is available in the eAppendix in the Supplement. For the primary analyses, we focused on patients in OLDW who would be eligible for the PRONOUNCE trial based on the operational definitions of the inclusion and exclusion criteria. One-to-one matching was used to balance the differences in baseline characteristics between patients who received degarelix vs those who received leuprolide. A PS, representing the probability of receiving degarelix, was estimated using a logistic regression model based on sociodemographic variables, medical history, concurrent medication use, and other patient characteristics presented in Table 1. One-to-one nearest-neighborhood caliper matching was used to match patients based on the logit of the PS, using a caliper equal to 0.2 of the SD of the logit of the PS.^33^ As patients with missing data were excluded during cohort selection, there were no missing data for any of the variables in the PS model expect for PSA level. Patients with and without PSA values were matched separately.

Standardized differences were used to assess the balance of covariates after matching, and a standardized difference 0.1 or smaller was considered acceptable.^34^ Covariates with standardized differences greater than 0.1 were adjusted for in the regression models.

Cox proportional hazards regression models were used to compare outcomes among patients who received degarelix with those who received leuprolide in the propensity-matched cohort, with robust sandwich estimates to account for the clustering within matched sets.^35^ The proportional hazard assumption was tested on the basis of Schoenfeld residuals.^36^ The Fine and Gray method was used to consider death as a competing risk when assessing nonfatal end points.^37^ All primary analyses compared the assigned treatment groups under the intention-to-treat (ITT) principle.

#### Subgroup and Sensitivity Analyses

We conducted subgroup (interaction) analyses by age, as outlined in the PRONOUNCE trial protocol. We also conducted subgroup analyses for important demographic and clinical characteristics: race, diabetes, presence of at least 1 prostate biopsy, and kidney function, using receipt of hemodialysis to identify patients with end-stage kidney disease. In addition, for the patients with laboratory data, we generated subgroups of patients with eGFRs of less than and at least 45 mL/min/ 1.73 m^2^.

We then performed several sensitivity analyses to assess the dependence of our results on our cohort definition and statistical approach. First, we repeated our analyses among patients with no previous history or current hormonal management of prostate cancer who failed to meet at least 1 of the cardiovascular inclusion criteria for PRONOUNCE and among patients who met at least 1 of the exclusion criteria. Next, we repeated our analyses excluding all patients who crossed over between the 2 treatments and censoring patients at the point at which they switched. Third, we repeated our primary ITT analyses using inverse probability of treatment weighting (IPTW)—average treatment effect weights—instead of PS matching to evaluate the consistency of our findings. Fourth, we conducted an analysis stratified by adherence to degarelix and leuprolide, ie, patients with proportion of days covered (PDC) of at least 80% and less than 80% until outcome date or the date that a patient switched medications. Lastly, we assessed residual confounding by testing 3 falsification end points, unlikely to be associated with use of degarelix or leuprolide: new diagnoses of chronic obstructive pulmonary disease (COPD), appendicitis, and cholecystitis, after the index date. All sensitivity analyses were considered exploratory.

*P* < .05 was considered statistically significant for all 2-sided tests. All analyses were performed using SAS version 9.4 (SAS Institute Inc) and Stata version 14.1 (StataCorp).

## Results

### Patient Characteristics

**Figure 1.**
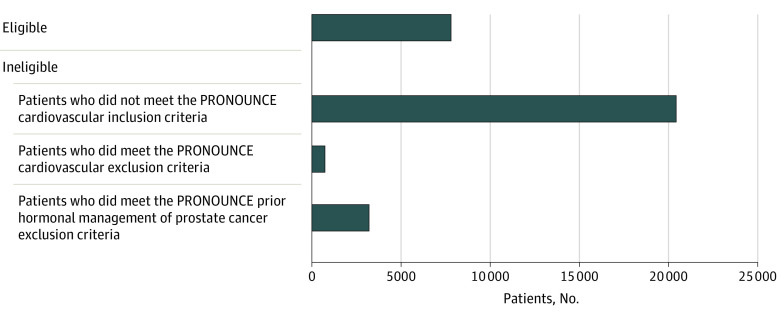
Patients Included and Excluded After Applying Inclusion and Exclusion Criteria From the PRONOUNCE Trial (a Trial Comparing Cardiovascular Safety of Degarelix Versus Leuprolide in Patients With Advanced Prostate Cancer and Cardiovascular Disease)

We identified 103 500 adult men who initiated degarelix or leuprolide between December 24, 2008, and June 30, 2019, of whom 32 172 had valid demographic data, 6 months of continuous enrollment before the index date, and a prostate cancer diagnosis (eFigure in the Supplement). PRONOUNCE trial eligibility criteria were met by 7800 of 32 172 patients (24.2%) (Figure 1; eTable 1 in the Supplement). Their mean (SD) age was 74.4 (7.4) years, and 165 participants (2.1%) were Asian, 1390 (17.8%) were Black, 663 (8.5%) were Hispanic, and 5258 (67.4%) were White. PSA level data were available for 2686 patients (34.4%), with a mean (SD) value of 53.0 (286.1) ng/mL (to convert to micrograms per liter, multiply by 1.0) (Table 1; eTable 2 in the Supplement). After PS matching, primary analyses included 1113 of 1120 patients (99.4%) treated with degarelix and 1113 of 6680 patients (16.7%) treated with leuprolide; these groups were well balanced with SMDs less than 0.10 for all 49 matching characteristics. For 1 year and 3 states, SMDs greater than 0.1 were observed, likely due to the low number of patients across the levels for these characteristics. However, in a sensitivity analysis adjusting for calendar year and state, the results remained the same.

### Primary ITT Analyses

**Figure 2.**
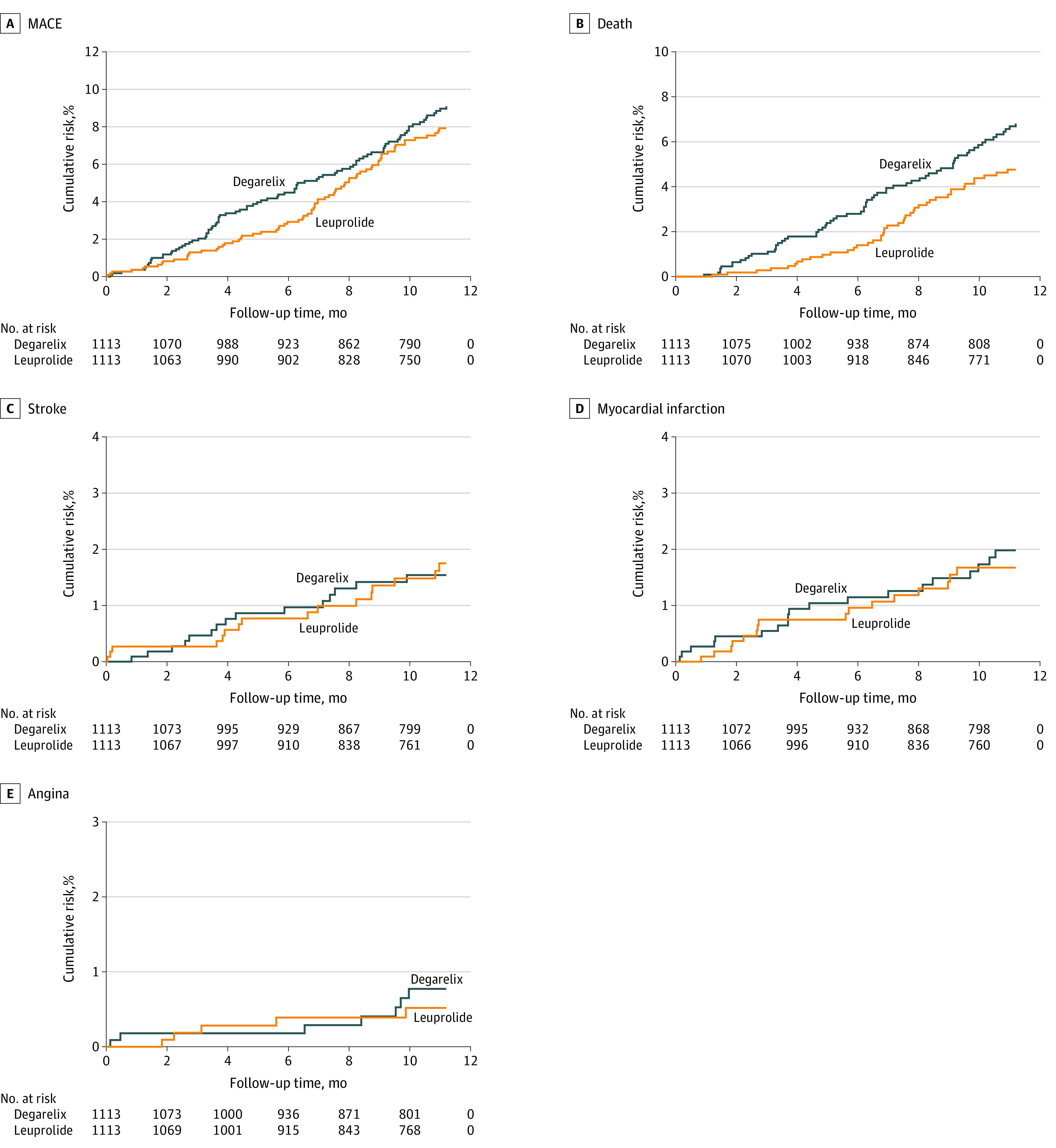
Cumulative Incidence of Major Adverse Cardiovascular Event (MACE), Death, Stroke, Myocardial Infarction, and Angina in the Overall Cohort MACE was a composite end point defined as death from any cause, nonfatal myocardial infarction, or nonfatal stroke.

Patients who initiated degarelix and leuprolide were observed in the data for a mean (SD) of 9.55 (2.98) months and 9.77 (2.74) months, respectively. No significant difference was observed in the risk of MACE for patients initiated on degarelix vs leuprolide (10.18 vs 8.60 events per 100 person-years; hazard ratio [HR], 1.18; 95% CI, 0.86-1.61; *P* = .30) (Table 2 and Figure 2). Degarelix was associated with a significantly higher risk of death from any cause (HR, 1.48; 95% CI, 1.01-2.18; *P* = .046) but not of myocardial infarction (HR, 1.16; 95% CI, 0.60-2.25; *P* = .66), stroke (HR, 0.92; 95% CI, 0.45-1.85; *P* = .81), or angina (HR, 1.36; 95% CI, 0.43-4.27; *P* = .60).

**Table 2.** End Points in 2226 Propensity-Matched Patients

End point	Degarelix (n = 1113)			Leuprolide (n = 1113)			Hazard ratio (95% CI)	*P* value
	Events, No.	Person-years	Event rate per 100 person-years	Events, No.	Person-years	Event rate per 100 person-years		
Primary end point								
MACE^a^	88	864.65	10.18	73	849.15	8.60	1.18 (0.86-1.61)	.30
Secondary end points								
Death	65	875.05	7.43	43	861.81	4.99	1.48 (1.01-2.18)	.046
Myocardial infarction	19	869.63	2.18	16	855.18	1.87	1.16 (0.60-2.25)	.66
Stroke	15	869.75	1.72	16	855.78	1.87	0.92 (0.45-1.85)	.81
Angina	<11^b^	NA	NA	<11^b^	NA	NA	1.36 (0.43-4.27)	.60

Abbreviations: MACE, major adverse cardiovascular event; NA, not applicable.

^a^ MACE was defined as a composite end point of death from any cause, myocardial infarction, or stroke.

^b^ Cell suppression based on OptumLabs cell size suppression rules. Categories with fewer than 11 individuals are masked to protect patient confidentiality.

### Subgroup and Sensitivity Analyses

There was no evidence of an interaction effect across subgroups for age, race, diabetes, end-stage kidney disease, and baseline eGFR (eTable 3 in the Supplement). Treatment effect sizes for the primary and secondary end points were largely consistent among the 2686 patients with a prostate biopsy and among the 2952 patients who failed to meet the cardiovascular inclusion criteria and hence would be excluded from PRONOUNCE (Table 3; eTable 4 in the Supplement).

**Table 3.** End Points in Sensitivity Analyses

Outcome	Degarelix				Leuprolide				Hazard ratio (95% CI)	*P* value
	Patients, No.	Events, No.	Person-years	Event rate, per 100 person-years	Patients, No.	Events, No.	Person-years	Event rate, per 100 person-years		
**Patients who failed to meet the cardiovascular inclusion criteria**										
MACE^a^	2952	112	2297.82	4.87	2952	92	2293.01	4.01	1.21 (0.92-1.60)	.17
Death	2952	83	2310.67	3.59	2952	54	2308.80	2.34	1.53 (1.09-2.16)	.02
Myocardial infarction	2952	12	2307.05	0.52	2952	23	2301.27	1.00	0.52 (0.26-1.04)	.06
Stroke	2952	27	2301.10	1.17	2952	27	2300.51	1.17	0.99 (0.58-1.70)	.98
Angina	2952	<11^b^	NA	NA	2952	<11^b^	NA	NA	0.66 (0.19-2.34)	.52
**Patients who met the cardiovascular exclusion criteria**										
MACE^a^	96	<11^b^	NA	NA	96	12	NA	NA	0.64 (0.27-1.56)	.33
Death	96	<11^b^	NA	NA	96	<11^b^	NA	NA	0.83 (0.29-2.44)	.74
Myocardial infarction	96	<11^b^	NA	NA	96	<11^b^	NA	NA	3.02 (0.32-28.87)	.34
Stroke	96	<11^b^	NA	NA	96	<11^b^	NA	NA	0.39 (0.08-2.00)	.26
Angina	96	<11^b^	NA	NA	96	<11^b^	NA	NA	NA	NA
**Patients who crossed over between 2 treatments censored when they switched**										
MACE^a^	1113	52	431.30	12.06	1113	72	847.11	8.50	1.58 (1.11-2.26)	.01
Death	1113	35	436.55	8.02	1113	42	859.76	4.89	1.95 (1.26-3.03)	.003
Myocardial infarction	1113	13	433.96	3.00	1113	16	853.14	1.88	1.57 (0.74-3.35)	.24
Stroke	1113	<11^b^	NA	NA	1113	16	NA	NA	1.10 (0.48-2.53)	.81
Angina	1113	<11^b^	NA	NA	1113	<11^b^	NA	NA	1.45 (0.39-5.43)	.58
**Patients who crossed over between treatments excluded**										
MACE^a^	398	47	289.85	16.22	398	32	310.44	10.31	1.58 (1.01-2.47)	.046
Death	398	35	294.34	11.89	398	17	314.92	5.40	2.21 (1.24-3.94)	.01
Myocardial infarction	398	<11^b^	NA	NA	398	14	310.58	4.51	0.66 (0.28-1.52)	.33
Stroke	398	<11^b^	NA	NA	398	<11^b^	NA	NA	1.67 (0.55-5.11)	.37
Angina	398	<11^b^	NA	NA	398	<11^b^	NA	NA	0.51 (0.09-2.79)	.44
**IPTW instead of propensity-score matching**										
MACE^a^	1120	593	6194.59	9.58	6680	531	6135.66	8.65	1.10 (0.86-1.41)	.43
Death	1120	460	6252.48	7.36	6680	304	6233.80	4.87	1.50 (1.13-2.01)	.01
Myocardial infarction	1120	132	6218.51	2.13	6680	139	6180.94	2.24	0.94 (0.56-1.58)	.82
Stroke	1120	89	6225.89	1.43	6680	128	6187.43	2.06	0.69 (0.38-1.24)	.21
Angina	1120	44	6235.09	0.71	6680	42	6214.76	0.68	1.03 (0.44-2.42)	.95
**PDC ≥80%**										
MACE^a^	690	50	523.12	9.56	690	52	494.69	10.51	0.90 (0.61-1.33)	.61
Death	690	36	530.74	6.78	690	31	505.73	6.13	1.09 (0.68-1.77)	.71
Myocardial infarction	690	13	526.60	2.47	690	15	498.44	3.01	0.83 (0.39-1.74)	.62
Stroke	690	<11^b^	NA	NA	690	<11^b^	NA	NA	1.07 (0.41-2.78)	.89
Angina	690	<11^b^	NA	NA	690	<11^b^	NA	NA	2.84 (0.58-13.99)	.20
**PDC <80%**										
MACE^a^	417	38	336.90	11.28	417	25	352.12	7.10	1.60 (0.97-2.64)	.07
Death	417	29	339.69	8.54	417	19	353.18	5.38	1.60 (0.90-2.85)	.11
Myocardial infarction	417	<11^b^	NA	NA	417	<11^b^	NA	NA	3.10 (0.63-15.60)	.16
Stroke	417	<11^b^	NA	NA	417	<11^b^	NA	NA	1.02 (0.33-3.17)	.97
Angina	417	<11^b^	NA	NA	417	<11^b^	NA	NA	0.34 (0.04-3.32)	.36

Abbreviations: IPTW, inverse probability of treatment weighting; MACE, major adverse cardiovascular event; NA, not applicable; PDC, proportion of days covered.

^a^ MACE was defined as a composite end point of death from any cause, myocardial infarction, or stroke.

^b^ Cell suppression based on OptumLabs cell size suppression rules. Categories with fewer than 11 individuals are masked to protect patient confidentiality.

We observed considerable crossover from degarelix to leuprolide, with a median (IQR) duration of degarelix exposure of 48 (33-92) days. There were 115 of 722 patients (15.9%) who crossed over within 30 days of initiating degarelix. When excluding patients who crossed over between the 2 treatments (722 who initiated degarelix and 19 who initiated leuprolide) or censoring those at the time of medication switch, degarelix was associated with higher risk of MACE (crossover: HR, 1.58; 95% CI, 1.01-2.47; *P* = .046; censoring: HR, 1.58; 95% CI, 1.11-2.26; *P* = .01) and death (crossover: HR, 2.21; 95% CI, 1.24-3.94; *P* = .01; censoring: HR, 1.95; 95% CI, 1.26-3.03; *P* = .003) (Table 3). There were no statistically significant associations between degarelix and myocardial infarction, stroke, and angina. The results of the IPTW analyses for MACE and death were consistent with the primary analyses.

No statistically significant differences between degarelix and leuprolide were observed across end points when patients were stratified by PDC of at least 80% vs less than 80%. There were also no significant associations between degarelix and any of the falsification end points of COPD, appendicitis, and cholecystitis (eTable 5 in the Supplement).

## Discussion

In a cohort of US patients with commercial insurance or Medicare Advantage and established cardiovascular disease initiating ADT for prostate cancer treatment, we emulated the forthcoming PRONOUNCE trial to examine cardiovascular risk of prostate cancer treatment. Using these RWD, we determined that only one-quarter of real-world patients who initiated these medications met the trial’s narrow inclusion and exclusion criteria. Among the men who did, we found no significant difference in the risk of MACE between patients taking degarelix vs those taking leuprolide. While we did observe degarelix to be associated with a significantly higher risk of death from any cause, which was consistent across a range of sensitivity analyses, there were no statistically significant differences between degarelix and leuprolide for myocardial infarction, stroke, or angina. Comparison of the patient characteristics and end points reported here with the PRONOUNCE trial results, upon their publication, will help to further enhance our understanding of the appropriate role of using RWD to emulate randomized clinical trials.

In contrast to previous studies, our findings do not support the hypothesis that degarelix is associated with a lower cardiovascular risk than leuprolide.^7,8,9,10,11,12,38^ Notably, no previous trial was designed with cardiovascular morbidity as the primary end point. Although degarelix was associated with an unexpectedly higher risk of MACE in the secondary analyses, this appears to be driven by the higher risk of all-cause mortality, as there was no difference in rates of MACE components, including myocardial infarction and stroke. It is likely that residual confounding due to patient and prescriber behaviors could have contributed to the observed findings, especially the increased risk of all-cause mortality, in ways that would not occur in an RCT. In particular, preferential use of degarelix for patients with more cardiovascular comorbidity and higher disease burden could not be risk-adjusted for using claims data. It is also possible that the inclusion of all-cause mortality in the MACE end point could have biased our results toward the null. Although we analyzed the components of MACE separately and matched the cohort on several cardiovascular baseline comorbidities, the presence of metastatic disease, and PSA levels, we could not account for all characteristics, and PSA data were not available for approximately two-thirds of patients. Overall, the impact of residual confounding on the direction and strength of the observed associations is unclear, and we await the results of the PRONOUNCE trial to ascertain whether the observations made here align with the trial’s enrolled patient population, outcome event rates, and inferences.

Our experience highlights several important considerations related to the interventions, eligibility criteria, end points, follow-up, and statistical analyses in designing RWD studies to emulate RCTs. First, emulation requires studying existing therapeutics and is not appropriate for novel drugs or therapies. Indeed, only 15% of US-based clinical trials published in high-impact journals in 2017 could have been evaluated using RWD.^18^ Second, adequate observational data are necessary to emulate trial eligibility criteria and evaluate end points. While we were able to emulate the major PRONOUNCE eligibility criteria, certain variables were not found in EHR data (eg, ankle-brachial pressure index).^18^ Similarly, precise emulation of end points—such as cancer-specific death or differentiation between fatal and nonfatal cardiovascular events—was not possible with claims data. Third, although we had adequate follow-up to emulate the PRONOUNCE trial, as our median follow-up period was 8 months vs 11 months for the trial, future studies should carefully consider the availability of enrollment dates to assess follow-up. For instance, emulation studies relying on EHR data would not have enrollment dates. Lastly, our experience demonstrates the analytical complexities of conducting emulation studies and implementing the ITT framework, particularly when patients cross over between interventions. These challenges emphasize the importance of sensitivity analyses to evaluate the consistency of results and conclusions.^27^ As the FDA continues to invest in the use of RWD to simulate evidence from clinical trials,^16^ it will be necessary for investigators to follow best practices when it comes to trial emulation (eg, the target trial framework).^27^ Moreover, it will be critical to compare concordant and discordant results between RCTs and RWD emulations, with a focus on understanding potential differences between patient characteristics and study results and identifying the best RWD structures, epidemiologic methods, and study characteristics suitable for emulation.

The methodologic approach applied here has a number of strengths, including some clear advantages over a traditional RCT and, if validated, may represent an ideal approach to help answer certain questions for existing therapies. First, our sample reflects actual clinical use of degarelix and leuprolide across a large cohort of patients over a 10-year period, and results are therefore generalizable to contemporary clinical practice. In particular, emulating RCTs using RWD can allow for the examination of benefits and risks in a broader population than any given clinical trial. Second, this emulation took approximately 1 year to design and conduct, a fraction of the time and cost of a traditional RCT.^14^ Third, evidence suggests that many of the clinical end points that we evaluated (ie, myocardial infarction, stroke, and death) are well captured in claims data.^39^ The available data allowed us to match patients on 49 different patient variables including comorbidities, procedures, and medications, and we anticipate the potential for even more refined risk adjustment in the future as the quality of claims data capture improves.

### Limitations

Despite the potential advantages of emulating RCTs using RWD, we acknowledge a number of limitations to this approach. First, despite PS matching and statistical adjustment, observational studies are subject to residual confounding. Second, we were unable to operationalize every component of certain inclusion and exclusion criterion and end points, as outlined previously. However, the sensitivity analyses and falsification end points provide some reassurance that there is no strong evidence for substantial residual confounding.^40^ Third, reliance on billing codes may have led to misclassification of medical history and end points. Fourth, we included patients who initiated additional androgen agents within 6 months after starting ADT, which could have affected the incidence of cardiovascular adverse events. However, meaningful confounding seems unlikely, as fewer than 10% of patients had additional hormonal agents after the index date. Fifth, we found evidence that relatively few patients started on degarelix without switching over to leuprolide in clinical practice and that exposure to degarelix was relatively short, which may bias our findings toward the null and adds an additional layer of complexity that would not have occurred in an RCT. While crossover likely occurred for patient convenience due to the less frequent dosing interval of leuprolide, it may have also occurred due to progressive disease, and thus, it may portend a greater risk of mortality, thereby influencing the primary MACE outcome. However, our findings were broadly consistent after excluding all patients who crossed over between the 2 treatments and censoring patients at the point at which they switched, increasing our confidence in the reported results. Sixth, our evaluation included patients who initiated degarelix or leuprolide between 2008 and 2019. However, since the PRONOUNCE Trial started in 2016, it will be necessary to ensure that the baseline characteristics between the real-world cohort and the trial population are similar before formally comparing the findings.

## Conclusions

In this emulated clinical trial of men with commercial insurance or Medicare Advantage in the United States who had cardiovascular disease and initiated ADT for prostate cancer, degarelix was not associated with a lower risk of cardiovascular events vs leuprolide, in contrast to prior reports. We observed frequent crossover between medications and missing data on disease characteristics in our cohort, which may have contributed to residual confounding and highlights some of the challenges of using RWD to emulate RCTs. The population included in this study as well as the findings should be compared with those of the PRONOUNCE trial, once available, to assess the fidelity of results from this real-world observational study with reported trial end points and to deepen our understanding of the optimal role of using RWD to emulate RCTs.

## Notes

Supplement. **eAppendix.** Study Protocol **eFigure.** Initial Cohort for PRONOUNCE **eTable 1.** Summary of Inclusion and Exclusion Criteria for PRONOUNCE Trial, Overall Cohort Population and Postcohort Development **eTable 2.** Use of Hormonal Management of Prostate Cancer and First-Generation Antiandrogen Agents Within 6 Months After Index Date and Baseline Characteristics Among Patients With and Without Prostate-Specific Antigen Levels **eTable 3.** Subgroup Analyses for the Primary Intention-to-Treat Propensity Score–Matched Analyses **eTable 4.** Outcomes in Additional Subgroup Analyses **eTable 5.** Falsification Analyses
