# Supplementary material for: Real-world Cardiovascular Outcomes Associated With Degarelix vs Leuprolide for Prostate Cancer Treatment
Source: JAMA Netw Open. 2021 Oct 22;4(10):e2130587. doi: 10.1001/jamanetworkopen.2021.30587 (PMC8536955; doi:10.1001/jamanetworkopen.2021.30587)
Supplement: Supplement. — eAppendix. Study Protocol eFigure. Initial Cohort for PRONOUNCE eTable 1. Summary of Inclusion and Exclusion Criteria for PRONOUNCE Trial, Overall Cohort Population and Postcohort Development eTable 2. Use of Hormonal Management of Prostate Cancer and First-Generation Antiandrogen Agents Within 6 Months After Index Date and Baseline Characteristics Among Patients With and Without Prostate-Specific Antigen Levels eTable 3. Subgroup Analyses for the Primary Intention-to-Treat Propensity Score–Matched Analyses eTable 4. Outcomes in Additional Subgroup Analyses eTable 5. Falsification Analyses [file jamanetwopen-e2130587-s001.pdf]

Supplemental Online Content

Wallach JD, Deng Y, McCoy RG, et al. Real-world cardiovascular outcomes associated with degarelix vs leuprolide for prostate cancer treatment. *JAMA Netw Open.* 2021;4(10):e2130587. doi:10.1001/jamanetworkopen.2021.30587

eAppendix. Study Protocol

eFigure. Initial Cohort for PRONOUNCE

eTable 1. Summary of Inclusion and Exclusion Criteria for PRONOUNCE Trial, Overall Cohort Population and Postcohort Development

eTable 2. Use of Hormonal Management of Prostate Cancer and First-Generation Antiandrogen Agents Within 6 Months After Index Date and Baseline Characteristics Among Patients With and Without Prostate-Specific Antigen Levels

eTable 3. Subgroup Analyses for the Primary Intention-to-Treat Propensity Score Matched Analyses

eTable 4. Outcomes in Additional Subgroup Analyses

eTable 5. Falsification Analyses

This supplemental material has been provided by the authors to give readers additional information about their work.

eAppendix. Study Protocol

The purpose of this analysis plan is to provide a detailed description of our methods and decisions, in order to guide our analyst(s). To facilitate replications of our methodology, the information in this document will also be shared in the supplementary materials of the final manuscript.

Protocol changes made after accessing the data are in bold.

1. ABBREVIATIONS

**ADT**	Androgen deprivation therapy
**ASCVD**	Atherosclerotic cardiovascular disease
**CABG**	Coronary artery bypass grafting
**CI**	Confidence interval
**CT**	Computed tomography
**DBP**	Diastolic blood pressure
**EHR**	Electronic health record
**E&M**	Evaluation & Management
**eGFR**	Estimated Glomerular Filtration Rate
**FDA**	Food and Drug Administration
**GnRH**	Gonadotropin-releasing hormone
**HbA1c**	Hemoglobin A1c
**HR**	Hazard ratio
**MACE**	Major Adverse Cardiovascular Events
**NA**	Not applicable
**PCI**	Percutaneous coronary intervention
**PDC**	Proportion of days covered
**PSA**	Prostate-specific antigen
**RCT**	Randomized controlled trials
**RWD**	Real world data
**SBP**	Systolic blood pressure
**SSA**	Social Security Administration

© 2021 Wallach JD et al. *JAMA Network Open.*

2. KEY DEFINITIONS

Item	Variable name	Description
**Index Date**	index_date	The date patients received degarelix (Firmagon) or leuprolide (Lupron depot)
**Index Medication**	index_med	Patient's first prescription of degarelix (Firmagon) or leuprolide (Lupron depot)
**Baseline Period**	baseline	Any time before and including the index date used to establish a patient's medical history
**Study Period**		12/24/2008 (degarelix (Firmagon) FDA approval date) – 6/30/2019
**Data Source**		OptumLabs Data Warehouse

3. BACKGROUND AND OBJECTIVES

Rigorous double-blind randomized controlled trials (RCTs) are considered the gold standard for generating evidence for the safety, efficacy, and effectiveness of any clinical intervention. Although RCTs are used to inform the decisions made by the Food and Drug Administration (FDA), health insurance payers, professional societies, patients, and clinicians,[1] these studies often have strict inclusion and exclusion criteria, small sample sizes, and short follow-up durations.[2] These limitations can undermine the generalizability of RCTs to real-world clinical practice, and have stimulated interested in research focused on generating evidence on medical products using observational research methods applied to real-world data sources.

Advances in the quantity, quality, and granularity of real-world data, and improvements in statistical methods used to account for unmeasured confounding, have provided opportunities to use real-world data to inform our understanding of drug safety and efficacy. While many studies have estimated the results of completed RCTs using real-world data and observational methods,[3-9] the majority have focused on comparisons with large prospective cohort studies, as opposed to retrospective analysis of routinely-collected electronic health record (EHR) or administrative claims data. Furthermore, less is known about the ability to simulate RCT eligibility criteria for ongoing trials to predict the characteristics of real-world populations and

© 2021 Wallach JD et al. *JAMA Network Open.*

determine whether the characteristics of the RCT population and the real-world populations are similar. Moreover, it would be valuable to predict the results of ongoing trials, thereby avoiding the potential biases that could be introduced by trying to replicate the results of RCTs that have already been completed and disseminated among the scientific community.

Using ClinicalTrials.gov, we have identified an illustrative test case to assess the potential opportunities, advantages, and limitations of using retrospective research methods to pursue evaluations of drug safety using real-world data, including as an alternative to RCTs. The PRONOUNCE trial is an ongoing Phase IIIb comparative safety trial focused on comparing the cardiovascular safety of degarelix (Firmagon), a gonadotropin-releasing hormone (GnRH) antagonist, and leuprolide (Lupron Depot), a GnRH agonist, among patients with prostate cancer and cardiovascular disease. Prostate cancer is the second most commonly occurring cancer in men, and among patients with advanced prostate cancer, androgen deprivation therapy (ADT) is a preferred treatment option.[10] However, studies have suggested that ADT in the form of GnRH agonists, such as leuprolide, increase the risk for cardiovascular morbidity and mortality, especially among prostate cancer patients with a history of cardiovascular disease.[11] Although GnRH antagonists are believed to be associated with improved cardiovascular safety compared to GnRH agonists,[12] the PRONOUNCE trial was designed to clarify uncertainties and compare the cardiovascular safety profile of the GnRH antagonist degarelix and the GnRH agonist leuprolide.

Using real-world data from OptumLabs, a large database of insurance claims linked with EHR data, we sought to predict the results of the PRONOUNCE trial by applying observational research methods to the real-world patients.

© 2021 Wallach JD et al. *JAMA Network Open.*

4. METHODS

4.1 Study design

This study will be a retrospective cohort analysis using OptumLabs Data Warehouse.

4.2 Data sources

OptumLabs Data Warehouse

OptumLabs Data Warehouse contains longitudinal health information on over 160 million predominantly privately insured individuals, and includes a large number of Medicare Part D and Medicare Advanced beneficiaries.[13] Patient-level information includes socio-demographic characteristics (i.e., age, sex, and race) and geographic region. Data also include health insurance coverage (dates of enrollment, primary/secondary coverage, Medicaid/Medicare status), administrative claims (diagnosis and procedure codes), and pharmacy claims (prescribing physician, pharmacy, days of supply, drug/quality/strength/data dispensed). These data also include physician & facility claims (physician type and specialty). For roughly 50 million individuals, additional information is available from electronic medical record encounters, including vital signs and laboratory measurements.

ClinicalTrials.gov and the PRONOUNCE trial Protocol

The PRONOUNCE trial was identified using ClinicalTrials.gov (**Box 1**). The trial inclusion and exclusion criterial were established using ClinicalTrials.gov and the descriptions outlined in the PRONOUNCE trial protocol (**Table 1**).[14]

**Box 1. PRONOUNCE trial Characteristics**
**Title:** A Trial Comparing Cardiovascular Safety of Degarelix Versus Leuprolide in Patients With Advanced Prostate Cancer and Cardiovascular Disease (PRONOUNCE)
**Aim(s):** To test whether degarelix (Firmagon), a marketed drug for advanced prostate cancer, can reduce the risk of cardiovascular complications compared to leuprolide (Lupron Depot), another drug for advanced prostate cancer, among patients with prostate cancer and cardiovascular disease.

© 2021 Wallach JD et al. *JAMA Network Open.*

**Primary endpoint:** Major Adverse Cardiovascular Events (MACE), including death due to any cause, non-fatal myocardial infarction, or non-fatal stroke.
**Secondary endpoints:** Time from randomization to occurrence of:<br>1. myocardial infarction (fatal, non-fatal)<br>2. stroke (fatal, non-fatal)<br>3. unstable angina requiring hospitalization (fatal, non-fatal)<br>4. cardiovascular-related death
Source: https://clinicaltrials.gov/ct2/show/NCT02663908?term=PRONOUNCE&draw=2&rank=1

Table 1. The PROUNCE Trial Eligibility Criteria

PRONOUNCE Eligibility Criteria	Operational Definition in OLDW
Inclusion Criteria	
Histologically confirmed adenocarcinoma of the prostate<br><br>Tumor, node, metastasis staging available prior to treatment start (bone scan and/or CT scan and/or MRI) <12 weeks prior to study start. If no radiographic image is available at the time of screening, a bone scan should be performed	NA: Already defined in the initial cohort as:<br><br>*Patients must have at least one "Evaluation and Management" visit with a diagnosis of prostate cancer within 6 months before index data, at least one "Evaluation and Management" visit with a diagnosis of prostate cancer at any time after the index date*<br><br>*Sensitivity analysis: prostate biopsy required*
Indication to initiate androgen deprivation therapy (ADT)	NA: 'Index date' is the first fill of degarelix or leuprolide*<br><br>**For the leuprolide cohort, we will allow up to one month of bicalutamide prior to leuprolide initiation*
**Predefined cardiovascular disease inclusion criteria**	
Pre-existing ASCVD (confirmed diagnosis, documented) according to at least one of the following criteria:<br><br>Prior myocardial infarction >=30 days before randomization; prior revascularization procedure >=30 days before randomization);<br>Coronary artery: stent placement/balloon angioplasty or coronary artery bypass graft surgery;<br>Coronary artery: stent placement/balloon angioplasty or endarterectomy surgery;<br>Iliac, femoral, popliteal arteries: stent placement/balloon angioplasty or vascular bypass surgery<br><br>At least one vascular stenosis >=50% at any time point before	To establish history of cardiovascular disease using claims data, it is common to use both primary and secondary discharge diagnosis and procedure codes. In particular, we will identify the following using discharge diagnosis and procedure codes as indicators of a history of cardiovascular disease, >= 30 days before the index date: Myocardial infarction, percutaneous coronary intervention (PCI), coronary artery bypass grafting (CABG), peripheral artery revascularization, carotid revascularization any position<br><br>*We do not have angiogram, CT angiogram, or ankle-brachial pressure data.*

© 2021 Wallach JD et al. *JAMA Network Open.*

randomization by angiogram or CT angiogram<br>Coronary artery<br>Carotid artery<br>Iliac femoral, or popliteal arteries<br>Carotid ultrasound results that documented a vascular stenosis >=50% at any time point before randomization<br><br>Ankle-brachial pressure index <0.9 at any time point before randomization.	
<u>Exclusion Criteria</u>	
Previous or current hormonal management of prostate cancer<br><br>- Surgical castration<br>- Any hormonal manipulation<br><br>Except prior neoadjuvant/adjuvant hormonal therapy, in this case treatment must be terminated >12 months prior to study start.	NA: 'Index date' is the first fill of degarelix or leuprolide<br><br>**For the leuprolide cohort, we will allow up to one month of bicalutamide prior to leuprolide initiation*
**Main cardiovascular exclusion criteria**	
Uncontrolled type 1 or type 2 diabetes mellitus (defined as HbA1c > 10%) at time of randomization	NA: Restricting patients with laboratory data would limit the sample
Uncontrolled hypertension (SBP >180 mmHg or DBP >110 mmHg) at time of randomization	NA: Restricting patients with laboratory data would limit the sample
A history of congenital long QT syndrome or risk factors for torsade de pointes ventricular arrhythmias (e.g., heart failure, hypokalemia, concomitant medication known to cause QT prolongation)	NA: This exclusion criteria only applies to patients at risk for ventricular arrhythmias
Within 30 days prior to randomization<br>- Myocardial infarction<br>- Stroke (hemorrhagic/ischemic)<br>- Coronary, carotid, or peripheral artery revascularization	To identify recent/active cardiovascular events, we will use primary diagnosis of myocardial infarction and stroke (emergency department or inpatient visits) within 30 days before index date, as patients hospitalized for an acute cardiovascular event would be expected to have these diagnoses listed as the primary discharge diagnosis, while patients with a history of cardiovascular disease would be expected to have these diagnoses listed as secondary discharge diagnoses.<br><br>Percutaneous coronary intervention (PCI), coronary artery bypass grafting (CABG), peripheral artery revascularization

© 2021 Wallach JD et al. *JAMA Network Open.*

	(limb events), carotid revascularization within 30 days before index date
Planned or scheduled cardiac surgery or PCI procedure that is known at the time of randomization	NA: We cannot determine planned or scheduled PCI procedures.
Ankle-brachial pressure index <0.9 at any point before randomization	NA: Restricting patients with laboratory data would limit the sample.
ASCVD = atherosclerotic cardiovascular disease; CT = computed tomography; DBP = diastolic blood pressure; HbA1c = hemoglobin A1c; NA = not applicable; SBP = systolic blood pressure;	

4.3 Study inclusion and exclusion criteria

We will apply the PRONOUNCE trial inclusion and exclusion criteria listed on ClinicalTrials.gov, which we will update with information made available by the PRONOUNCE trial authors,[14] to patients represented in OptumLabs data (**Table 1**). We will not restrict the real-world sample to the planned sample size for the trial, but rather include all patients who otherwise meet the eligibility criteria. However, we will apply successive inclusion, and then exclusion criteria, determining which criteria have the biggest impact on the size of the population of patients observed in real-world data (**Table 2**).

The PRONOUNCE trial includes male patients, without any age restrictions, with advanced prostate cancer and cardiovascular disease, who were treated with degarelix (Firmagon) or leuprolide (Lupron depot) (**Table 3**). We will first identify all patients who initiated degarelix and leuprolide between 12/24/2008 and 6/30/2019. The start date was selected because degarelix received FDA approval on 12/24/2008. The date of an individual's first treatment (first fill date) with degarelix or leuprolide will be defined as the index date.

**Table 2. Impact of inclusion/exclusion criteria**				
**PRONOUNCE Eligibility Criteria**	**Operational Definition in OLDW**	**Overall Cohort (N=)**	**Degarelix (Firmagon) (N =)**	**Leuprolide (Lupron Depot) (N =)**

© 2021 Wallach JD et al. *JAMA Network Open.*

Total % of patients eligible for PRONOUNCE				
Total % of patients ineligible for PRONOUNCE				
**Patients who met one of the following exclusion criteria (%)**				
Previous or current hormonal management of prostate cancer	A prescription fill of ADT medications within 6 months before the index date *OR* Procedure codes for bilateral orchiectomy within 6 months before the index date			
Within 30 days prior to randomization:<br>-Myocardial infarction<br>-Stroke (hemorrhagic)<br>-Coronary, carotid, or peripheral artery revascularization	Primary diagnosis of myocardial infarction and stroke (emergency department or inpatient visits) within 30 days before index date; percutaneous coronary intervention, coronary artery bypass grafting, peripheral artery revascularization (limb events), carotid revascularization within 30 days before index date			

Table 3. Generic Names of Medical Therapy

Brand name	Drug class	Generic Names
**Firmagon**	Gonadotropin-releasing hormone receptor antagonists	Degarelix
**Lupron Depot**	Gonadotropin-releasing hormone receptor agonist	Leuprolide, leuprolide acetate

We will then identify all male enrollees, without any age restrictions, with valid demographic (age and race/ethnicity) and residence data. All enrollees will be required to have at least 6 months of continuous enrollment with medical and pharmacy coverage (i.e. no more than 45 days gap in coverage) before the index date, in order to capture an adequate prior medical history.

We developed an algorithm to identify enrollees with prostate cancer based on clinical expertise and similar methodology outlined in previous studies, which reported positive

predictive values between 70% and 82%.[15-18] Specifically, we will require patients to have at least one Evaluation and Management (E&M) visit with a diagnosis of prostate cancer within 6 months before the index date and at least one E&M visit with a diagnosis of prostate cancer any time after the index date. We are not able to ascertain prostate cancer severity, so no categorization by prostate cancer grade will be possible. For our primary analysis, a prostate biopsy will not be required, since patients may receive a diagnosis from a biopsy of metastatic site (e.g. bone or lymph node). As a secondary analysis, we will restrict to a subcohort of patients with at least one prostate biopsy.

To identify patients with a history of cardiovascular disease, it common to use both primary and secondary discharge diagnosis and procedure codes. This is an established method for administrative claims data research and used extensively for cohort creation and quality measurement by the Centers for Medicare & Medicaid Services (CMS).[19] In particular, we will identify the following using discharge diagnosis and procedure codes as indicators of a history of cardiovascular disease, at least 30 days before the index date: myocardial infarction, percutaneous coronary intervention (PCI), coronary artery bypass grafting (CABG), peripheral artery revascularization, carotid revascularization any position.

Among patients with prostate cancer and pre-defined cardiovascular disease, we will use pharmacy claims data to exclude patients with a prescription fill of ADT medications within 6 months before the index date. However, for the leuprolide cohort, we will allow patient to remain eligible for inclusion even if they received bicalutamide within one month prior to leuprolide initiation. Leuprolide, a GnRH agonist, can paradoxically lead to a transient increase in testosterone during the first 1 to 3 weeks of treatment. Therefore, bicalutamide is often given for a few weeks before the initial leuprolide injection in order to block any potential adverse effects from the testosterone flare. Degarelix, a GnRH antagonist, does not produce a testosterone flare.

© 2021 Wallach JD et al. *JAMA Network Open.*

Lastly, we will exclude patients with recent/active cardiovascular events. We will use primary diagnosis of myocardial infarction and stroke (emergency department or inpatient visits) within 30 days before index date, as patients hospitalized for an acute cardiovascular event would be expected to have these diagnoses listed as the primary discharge diagnosis, while patients with a history of cardiovascular disease would be expected to have these diagnoses listed as secondary discharge diagnoses.

5. Measurements

5.1 Baseline characteristics

We will record and summarize key baseline characteristics, including socio-demographic characteristics, comorbidities, and prior and concurrent medication use (**Table 4**). Socio-demographic characteristics include age (mean, median, categories (<=54, 55-64, 65-74, 75+), and race/ethnicity (Asian, Black, Hispanic, White, Unknown). The race/ethnicity data provided by OptumLabs is primarily self-reported, with imputation by the data provider based on other available administrative data when it is missing.[20]

Update August 2020: After accessing the data and running preliminary analyses, we updated our protocol to account for additional baseline comorbidities (*italicized and underlined* in Table 4). These comorbidities were selected to account for residual confounding by severity of disease. In particular, we observed that degarelix was paradoxically associated with increased mortality. To further account for observed imbalances between the degarelix and leuprolide patients, we also matched on state.

Medical history will be determined using patients' physician, facility, and pharmacy claims before or on the index date. We will use all data available to us to establish patients' medical history (**Table 4**). Data from the baseline 6-months period will be used for all covariates, unless otherwise specified. In OptumLabs, approximately one third of patients with insurance claims data have linked laboratory results, and the availability depends on the contracts

between labs testing facilities and the OptumLabs Data Warehouse. For the patients with laboratory data, we will determine serum prostate-specific antigen (PSA) levels and estimated Glomerular Filtration Rate (eGFR). We will also determine whether patients had a prostate biopsy or received radiotherapy within 6 months before the index date. Previous treatment with bicalutamide and other baseline medications will be determined 6 months prior to index date.

Table 4. Baseline characteristics before and after propensity score matching[a]								
	Before PS Matching				After PS Matching			
	Degarelix (N=)	Leuprolide (N=)	Total (N=)	SMD	Degarelix (N=)	Leuprolide (N=)	Total (N=)	SMD
**Age**								
Mean (SD)								
Median (IQR)								
**Age group**								
<=54								
55-64								
65-74								
75+								
**Race/Ethnicity**								
Asian								
Black								
Hispanic								
White								
Unknown								
**Geographic Region**								
Midwest								
Northeast								
South								
West								
Unknown								
**Serum PSA level**								
N								
Mean (SD)								
Median (IQR)								
**Estimated Glomerular Filtration Rate (eFGR)**								
Number								
Mean (SD)								
Median (IQR)								

© 2021 Wallach JD et al. *JAMA Network Open.*

Table 4. Baseline characteristics before and after propensity score matching[a]								
	Before PS Matching				After PS Matching			
	Degarelix (N=)	Leuprolide (N=)	Total (N=)	SMD	Degarelix (N=)	Leuprolide (N=)	Total (N=)	SMD
**Prior prostate biopsy (within 6 months of index date)**								
**Baseline comorbidities**								
Coronary artery disease								
Chronic kidney disease								
Congestive heart failure								
Cerebrovascular disease								
Peripheral vascular disease								
Obesity								
Atrial Fibrillation								
Sleep apnea								
Hypertension								
MI								
Stroke								
PCI								
CABG								
PAD								
Dementia								
COPD								
Peptic ulcer disease								
Mild liver disease								
Diabetes without chronic complication								
Diabetes with chronic complication								
Metastatic solid tumor								
Rheumatic disease								
Charlson comorbidity score								
Mean (SD)								
Median (IQR)								

© 2021 Wallach JD et al. *JAMA Network Open.*

Table 4. Baseline characteristics before and after propensity score matching[a]								
	Before PS Matching				After PS Matching			
	Degarelix (N=)	Leuprolide (N=)	Total (N=)	SMD	Degarelix (N=)	Leuprolide (N=)	Total (N=)	SMD
**Prior radiotherapy (within 6 months before index date)**								
**Prior use of bicalutamide (within 6 months before index date)**								
**Other baseline medications (within 6 months before index date)**								
Statin								
Non-statin lipid lower medications								
ACEi								
ARB								
ACEi/ARB								
Sacubitril / Valsartan								
Warfarin								
DOAC								
Beta-blockers								
Loop diuretics								
Aldosterone antagonist								
Digoxin								
Calcium channel blocker								
Antiplatelet								
**Number of hospitalizations**								
0								
1								
2+								
**Number of ER visits**								
0								
1								
2+								
**Year of Cohort Entry**								
2008								
2009								
2010								
2011								

© 2021 Wallach JD et al. *JAMA Network Open.*

Table 4. Baseline characteristics before and after propensity score matching[a]								
	Before PS Matching				After PS Matching			
	Degarelix (N=)	Leuprolide (N=)	Total (N=)	SMD	Degarelix (N=)	Leuprolide (N=)	Total (N=)	SMD
2012								
2013								
2014								
2015								
2016								
2017								
2018								
2019								
State								
AL								
AZ								
CA								
CT								
FL								
GA								
IL								
IN								
MA								
MN								
MO								
NC								
NJ								
NY								
OH								
RI								
SC								
TN								
TX								
UT								
VA								
WI								
OTHER								

ACEi, angiotensin-converting-enzyme inhibitors; ARB, angiotensin II receptor blocker; CABG, coronary artery bypass grafting; COPD, chronic obstructive pulmonary disease; DOAC, direct-acting oral anticoagulants; IQR, interquartile range; MI = myocardial infarction; PAD, peripheral artery disease; PCI, percutaneous coronary intervention

[a] Underlined values were added after data were accessed

5.2 Follow-up and outcome ascertainment

OptumLabs Data Warehouse is continuously updated on a monthly basis and the data are complete within 6 months of the service being provided. The analyses of this study will be

© 2021 Wallach JD et al. *JAMA Network Open.*

performed from May to September 2020, implying that the most recent data available to us will be up to October, 2019. Therefore, patients will be followed until the end of the study period (07/31/2019), the end of enrollment in health insurance plans, or death, whichever is first.

5.3 Study outcomes

We will use similar primary and secondary endpoints as the PRONOUNCE trial (**Box 1**). The primary endpoint in the PRONOUNCE trial is the time to first occurrence of the composite Major Adverse Cardiovascular Event (MACE) endpoint, defined as death due to any cause, non-fatal myocardial infarction, or non-fatal stroke. The secondary endpoints in the PRONOUNCE trial include: time from randomization to occurrence of fatal and non-fatal myocardial infarction, fatal and non-fatal stroke, fatal and non-fatal unstable angina requiring hospitalization, and cardiovascular-related death as separate outcomes. Using OptumLabs data, we are able to determine stroke, myocardial infarction, and angina, but are unable to distinguish between fatal and non-fatal events (**Table 5**). However, we will use commonly used, published, and previously validated diagnosis and procedure codes for MACE. For instance, previous evaluations suggest that the performance of similar MACE outcome codes are relatively good, with positive predictive values between 88.4% and 94% for myocardial infarction, 85% for ischemic stroke, and 80%-98% for hemorrhagic stroke.[21-25]

© 2021 Wallach JD et al. *JAMA Network Open.*

<table>
<tr><th colspan="4">Table 5. PRONOUNCE trial Endpoint Definition</th></tr>
<tr><th></th><th>PRONOUNCE trial Definition</th><th>Operational Definition in OLDW</th><th>Differences and ICD codes</th></tr>
<tr><td>Primary endpoint</td><td>Time from randomization to the first confirmed (adjudicated) occurrence of the composite major adverse cardiovascular event (MACE) endpoint, defined as death due to any cause, non-fatal myocardial infarction or non-fatal stroke [up to 336 days]</td><td>Time from randomization to the first confirmed (adjudicated) occurrence of the composite Major Adverse Cardiovascular Event (MACE) endpoint, defined as death due to any cause, myocardial infarction, or stroke [up to 336 days]</td><td rowspan="3">In OLDW, we are unable to distinguish between fatal and nonfatal events.

Myocardial infarction:
ICD-9: 43301, 43311, 43321, 43331, 43381, 43391, 43401, 43411, 43491, 436, 430, 431, 444X, 435X
ICD-10: I693, I63X

Stroke:
ICD-9: 43301, 43311, 43321, 43331, 43381, 43391, 43401, 43411, 43491, 436, 430, 431, 444X, 435X
ICD-10: I693, I63X</td></tr>
<tr><td rowspan="4">Secondary endpoints</td><td>Time from randomization to occurrence of myocardial infarction (fatal, non-fatal) [up to 336 days]</td><td>Time from randomization to occurrence of myocardial infarction [up to 336 days]</td></tr>
<tr><td>Time from randomization to occurrence of stroke (fatal, non-fatal) [up to 336 days]</td><td>Time from randomization to occurrence of stroke [up to 336 days]</td></tr>
<tr><td>Time from randomization to occurrence of unstable angina requiring hospitalization (fatal, non-fatal) [up to 336 days]</td><td>Time from randomization to occurrence of angina [up to 336 days]</td><td>ICD-9: 413X
ICD-10: I20X</td></tr>
<tr><td>Time from randomization to occurrence of cardiovascular-related death [up to 336 days]</td><td>Time from randomization to occurrence of all-cause mortality [up to 336 days]</td><td>In OLDW, we are unable to distinguish between fatal and non-fatal events.</td></tr>
<tr><td colspan="4">ICD = International Classification of Diseases, OLDW = OptumLabs Data Warehouse</td></tr>
</table>

Mortality will be identified based on the Social Security Death Master File and discharge status. Before November 2011, the Social Security Death Master File has complete mortality

data. However, effective on November 1st, 2011, Section 205(r) of the Social Security Act prohibits the Social Security Administration (SSA) from disclosing state death records that SSA receives through its contracts with the states, except in limited circumstances. Thus, if the SSA knows of a death only from the states and not from any of its other sources of death information, which happens roughly one-third of the time, those death data will not appear on the Death Master File.[26] Using discharge status (i.e. in-hospital death), we typically capture an additional 30% of deaths beyond what has been captured by Death Master File; we anticipate therefore that most of the deaths missing from Death Master File should be captured by discharge status, particularly since most deaths occur in an institutional setting. We acknowledge that a small proportion of patients who died out of hospital and were not captured by Death Master File could be missing, however, this should be non-differential between treatment groups and should not influence our comparison.

5.4 Study follow-up

For each patient, we will also determine the follow-up time, which will start the day after initiation of degarelix or leuprolide. Follow-up will continue until the date when the patient experiences any of the following events:

(a) An outcome of interest

(b) End of insurance coverage (end of patient enrollment)

(c) Death

(d) Reaches the maximum anticipated follow-up of the trial (336 days)

5.5 Missing data

Patients will be considered to have a condition, comorbidity, outcome, or drug exposure if they have a corresponding claim, and will be considered not having a comorbidity, outcome or drug exposure if they do not have a corresponding claim. Although we will therefore not have

© 2021 Wallach JD et al. *JAMA Network Open.*

missing comorbidities, drug use, or outcomes data, misclassification may exist. While this is a limitation of using claims data, the algorithms used to define our inclusion/exclusion criteria, outcomes of interest, and important covariates are commonly used and have demonstrated good performance in previous studies. We suspect that any existing misclassification will be unrelated to treatment group and should not meaningfully impact our findings.

We will exclude patients with invalid demographic data during the cohort creation process (e.g., missing residence region or inconsistent birth year). However, we anticipate fewer than 1% of patients being excluded during the cohort creation. For race/ethnicity, the categories in the database are non-Hispanic white, non-Hispanic black, Hispanic, Asian, other and unknown. The other and unknown will be used as a separate category in the propensity score model.

6. STATISTICAL METHODS

6.1 Main analysis using OptumLabs cohort

For our primary analyses, we will focus on OptumLabs patients who would be eligible for PRONOUNCE based on the operational definitions of the inclusion and exclusion criteria in **Table 1** (base population)**.**

Propensity score matching will be used to balance the difference in baseline characteristics between patients who received degarelix versus those who received leuprolide. A propensity score, the probability of receiving degarelix, will be estimated using a logistic regression model which includes patient characteristics presented in **Table 4.** No interaction terms will be used. One-to-one nearest neighborhood caliper matching will be used to match patients based on the logit of the propensity score using a caliper equal to 0.2 of the standard deviation of the logit of the propensity score.[27] Standardized differences will be used to assess the balance of covariates after matching and a standardized difference within 0.1 will be

considered acceptable.[28] Covariates with standardized differences above 0.1 will be adjusted for in the regression models.

Cox proportional hazards regression will be used to compare patients receiving degarelix versus those who received leuprolide for the primary and secondary outcomes in the propensity matched cohort, with robust sandwich estimates to account for the clustering within matched sets.[29] The proportional hazard assumption will be tested on the basis of Schoenfeld residuals.[30] If the proportional hazard assumption is not met, we will assess alternative time to event models, including parametric models, using Akaike information criterion and Bayesian information criterion to determine the final model specification. The Fine and Gray method will be used to consider death as a competing risk when assessing non-fatal outcomes.[31] All primary analyses will compare the assigned treatment groups under the intention-to-treat principle.

All analyses will be conducted using SAS 9.4 (SAS Institute Inc., Cary, North Carolina) and Stata 16 (Stata Corp, College Station, TX).

6.2 Subgroup Analyses

First, we will repeat our analyses restricted to a subcohort of patients with at least one prostate biopsy. Next, we will perform subgroup analyses for the primary outcome stratified by age, race, diabetes mellitus, and renal function, using receipt of hemodialysis to identify patients with end-stage renal disease. In addition, for the patients with laboratory data, we will generate subgroups of patients with eGFR <45 and >45. The subgroup analyses will be performed separately in patients who were eligible for the trial (primary analysis) and patients who failed to meet the inclusion criterion/exclusion criteria (secondary analyses). Within each subgroup, we will re-examine the standardized differences to assess the balance of the covariates. If the majority of the standardized differences are above 0.01, we will rematch the patients within each subgroup. Since an increasing number of subgroup analyses could increase the chance of false

positive results, we pre-specified the above subgroups since they are either key demographic characteristics or risk factors strongly associated with the primary outcome. However, we will not perform any adjustment for multiple testing.

6.3 Sensitivity Analyses

We will conduct the following sensitivity analyses to assess the robustness of the findings:

1. We will repeat our analyses across two subgroups: (1) patients who failed to meet any one of the cardiovascular inclusion criteria for PRONOUNCE; and (2) patients who met at least one of the cardiovascular exclusion criteria. These subgroups are of particular interest because they represent patient populations that may have been eligible for degarelix or leuprolide treatment, outside of the stringent inclusion/exclusion criteria used by clinical trials. In particular, patients in subgroup 1 are likely to have lower event rates and patients in subgroup 2 are likely to be at higher risk and bad candidates for new drugs. Some patients may have both failed to meet the inclusion criteria and met the exclusion criteria. In the stratified analyses for clinical outcomes, such patients will be classified as those who met the exclusion criteria. Second
2. **Update August 2020: After accessing the data and running preliminary analyses, we discovered that there was cross-over between the two treatments. Therefore, we added three sensitivity analyses to account for the cross-over: Leuprolide, a GnRH agonist, can paradoxically lead to a transient increase in testosterone during the first 1 to 3 weeks of treatment. Therefore, degarelix, a GnRH antagonist that does not produce a testosterone flare and requires fewer office visits, can be given prior to leuprolide. To account for the possibility of patients crossing-over between the two treatment groups, we will repeat our analyses: (1) excluding all patients that cross-over between the two treatments and (2) censoring patients at**

© 2021 Wallach JD et al. *JAMA Network Open.*

<u>the point at which they switch. We used inverse probability of treatment weighting (IPTW) instead of propensity score matching to minimize confounding.</u>

3. We will conduct a stratified analysis based on the adherence to degarelix and leuprolide, i.e., patients with proportion of days covered (PDC)<80% and those with PDC≥80%, since the adherence to medical therapy in practice is often lower than that in clinical trials. The adherence will consider all drugs that a patient used during follow up, even if they were different from the initial treatment. For degarelix, which is administered monthly, we will use 30 days supply. For leuprolide, there are multiple dosing intervals, which makes timing of the next dose dependent on the dose given at the last injection. For leuprolide patients without dose information, we will assume that fills were for 30 days. After matching the degarelix patients with PDC≥80% with the leuprolide patients with PDC<80%, we will conduct the cox regression analyses to compare the outcomes between the two groups. Analyses will be repeated among patients with PDC<80%.
4. We will assess falsification endpoints to test for residual confounding. Treatment effects estimated in observational studies are prone to unmeasured confounding. In recent years, a falsification end point, also called a control outcome, has become a popular method to assess for unmeasured confounding.[32-34] A falsification endpoint is a health outcome that researchers believe is highly unlikely to be casually related to the treatment in question. If a significant relationship is found between the treatment and a falsification endpoint, it may indicate the treatment groups are different in some unmeasured ways, i.e. the existence of unmeasured confounding. This method is similar to a negative control, a routine precaution taken in the design of biologic laboratory experiments, and is recommended to be used to detect confounding and bias in observational studies.[33,35,36] This method is particularly useful in observational studies comparing different treatment options, because the unmeasured confounding in these

© 2021 Wallach JD et al. *JAMA Network Open.*

studies tend to make one group systematically healthier or less susceptible to adverse outcomes than the other group.

We selected two endpoints that that are unlikely to be associated with use of either degarelix or leuprolide – chronic obstructive pulmonary disease and appendicitis/cholecystitis. If a significant relationship were to be found between degarelix and any of these endpoints, it would indicate the existence of residual confounding.

6.4 Comparison of cohort and trial population characteristics and results

Once the ongoing trials have been completed, we will compare the trial population to the population of patients identified in the claims component of OptumLabs data after application of the pre-specified eligibility criteria, as described above, to determine how accurately the characteristics of trial populations can be predicted. For each individual characteristic included in **Table 4** and reported in the PRONOUNCE trial publication, we will make pairwise comparisons between the trial population and the population of patients identified in the claims component of OptumLabs, stratified by treatment arm. In particular, within the degarelix and leuprolide arms, we will take the paired differences between the standardized mean differences (Cohen's d) from the trial population and the real-world population. Differences between standardized mean differences within 0.2 will be considered acceptable.

We will also compare rates of missing data and loss to follow-up across arms. If we observe significant differences in the characteristics of the real-world and trial populations, we will estimate a real-world population reweighted to mirror the characteristics of the PRONOUNCE trial population.

Once the PRONOUNCE trial have been completed and published, we will compare the final primary and secondary endpoint results to the results estimated from A) the population of real-world patients meeting pre-specified trial eligibility criteria and (when needed as explained above) B) the population of real-world patients reweighted to mirror the characteristics of the

final enrolled population of patients in the trial, for each analytic approach employed as described above. As needed, we will also compare each of the observational approaches used above to the RCT results, providing a better understanding of the tradeoffs inherent to each of the proposed methods.

Two approaches for comparing results will be used. First, as a simple method, results from both the real-world data and the trial will be characterized as positive (i.e., degarelix statistically significantly reduces the risk of cardiovascular complications as compared to leuprolide), neutral (no statistically significant difference between degarelix and leuprolide), or negative (degarelix statistically significantly increases the risk of cardiovascular complications as compared to leuprolide) and a percent agreement will be estimated. Statistical tests will be 2-sided and significance will be set at $P < 0.05$.

Second, we will pursue a more sophisticated method. The hazard ratios calculated for the primary and secondary outcomes using the real-world data will be converted to natural logarithm hazard ratios (lnHR). For each outcome, we will then take the difference between the lnHR calculated using the real-world data and the lnHR reported by the PRONOUNCE trial. After exponentiating each difference, a ratio of hazard ratios greater than 1.0 will imply greater (more beneficial) treatment effects in the real-world population than in the PRONOUNCE trial population. We will calculate 95% confidence intervals for the ratios of the hazard ratios by taking the square root of the sum of the variance for the hazard ratio derived from the real-world data and the variance for the hazard ratio from the PRONOUNCE trial population. Our variance calculations will be based on assumption of independence (i.e. correlation coefficients of zero, indicating that the outcomes from the real-world data and trial data are independent).[37]

© 2021 Wallach JD et al. *JAMA Network Open.*

REFERENCES

1. Office of Technology Assessment, U.S. Congress. The Impact of Randomized Clinical Trials on Health Policy and Medical Practice: Back-ground paper (OTA-BP-H-22). August 1983: https://repository.library.georgetown.edu/bitstream/handle/10822/708483/8310.PDF?sequence=1.Accessed July 26, 2018. In.
2. Sherman RE, Anderson SA, Dal Pan GJ, et al. Real-World Evidence - What Is It and What Can It Tell Us? *N Engl J Med.* 2016;375(23):2293-2297.
3. Hernán MA, Robins JM. Estimating causal effects from epidemiological data. *J Epidemiol Community Health.* 2006;60(7):578-586.
4. Melloni C, Washam JB, Jones WS, et al. Conflicting results between randomized trials and observational studies on the impact of proton pump inhibitors on cardiovascular events when coadministered with dual antiplatelet therapy: systematic review. *Circ Cardiovasc Qual Outcomes.* 2015;8(1):47-55.
5. Fralick M, Kesselheim AS, Avorn J, Schneeweiss S. Use of Health Care Databases to Support Supplemental Indications of Approved Medications. *JAMA Intern Med.* 2018;178(1):55-63.
6. Lonjon G, Boutron I, Trinquart L, et al. Comparison of treatment effect estimates from prospective nonrandomized studies with propensity score analysis and randomized controlled trials of surgical procedures. *Ann Surg.* 2014;259(1):18-25.
7. Fretheim A, Zhang F, Ross-Degnan D, et al. A reanalysis of cluster randomized trials showed interrupted time-series studies were valuable in health system evaluation. *J Clin Epidemiol.* 2015;68(3):324-333.
8. Tannen RL, Weiner MG, Xie D, Barnhart K. A simulation using data from a primary care practice database closely replicated the women's health initiative trial. *J Clin Epidemiol.* 2007;60(7):686-695.
9. Tannen RL, Weiner MG, Xie D. Replicated studies of two randomized trials of angiotensin-converting enzyme inhibitors: further empiric validation of the 'prior event rate ratio' to adjust for unmeasured confounding by indication. *Pharmacoepidemiol Drug Saf.* 2008;17(7):671-685.
10. Dawson NA. Patient education: Treatment for advanced prostate cancer (Beyond the Basics). In: UpToDate, Post TW (Ed), UpToDate, waltham, MA. (Accessed on 3 April, 2020). In.
11. Nanda A, Chen MH, Braccioforte MH, Moran BJ, D'Amico AV. Hormonal therapy use for prostate cancer and mortality in men with coronary artery disease-induced congestive heart failure or myocardial infarction. *JAMA.* 2009;302(8):866-873.
12. Albertsen PC, Klotz L, Tombal B, Grady J, Olesen TK, Nilsson J. Cardiovascular morbidity associated with gonadotropin releasing hormone agonists and an antagonist. *Eur Urol.* 2014;65(3):565-573.
13. Wallace PJ, Shah ND, Dennen T, Bleicher PA, Bleicher PD, Crown WH. Optum Labs: building a novel node in the learning health care system. *Health Aff (Millwood).* 2014;33(7):1187-1194.
14. Melloni C, Solvin SF, Blemings A, et al. Cardiovascular Safety of Degarelix Versus Leuprolide for Advanced Prostate Cancer: The PRONOUNCE Trial Study Design. 2020;2(1).
15. Unger JM, Hershman DL, Till C, et al. Using Medicare Claims to Examine Long-term Prostate Cancer Risk of Finasteride in the Prostate Cancer Prevention Trial. *J Natl Cancer Inst.* 2018;110(11):1208-1215.

© 2021 Wallach JD et al. *JAMA Network Open.*

16. McClish DK, Penberthy L, Whittemore M, et al. Ability of Medicare claims data and cancer registries to identify cancer cases and treatment. *Am J Epidemiol.* 1997;145(3):227-233.
17. Nordstrom BL, Whyte JL, Stolar M, Mercaldi C, Kallich JD. Identification of metastatic cancer in claims data. *Pharmacoepidemiol Drug Saf.* 2012;21 Suppl 2:21-28.
18. Cooper GS, Yuan Z, Stange KC, Dennis LK, Amini SB, Rimm AA. The sensitivity of Medicare claims data for case ascertainment of six common cancers. *Med Care.* 1999;37(5):436-444.
19. Centers for Medicare & Medicaid Services. Measures Methodology. https://www.cms.gov/Medicare/Quality-Initiatives-Patient-Assessment-Instruments/HospitalQualityInits/Measure-Methodology. Accessed 17 July 2020. In.
20. Hershman DL, Tsui J, Wright JD, Coromilas EJ, Tsai WY, Neugut AI. Household net worth, racial disparities, and hormonal therapy adherence among women with early-stage breast cancer. *Journal of Clinical Oncology.* 2015;33(9):1053-1059.
21. Kiyota Y, Schneeweiss S, Glynn RJ, Cannuscio CC, Avorn J, Solomon DH. Accuracy of Medicare claims-based diagnosis of acute myocardial infarction: estimating positive predictive value on the basis of review of hospital records. *Am Heart J.* 2004;148(1):99-104.
22. Wahl PM, Rodgers K, Schneeweiss S, et al. Validation of claims-based diagnostic and procedure codes for cardiovascular and gastrointestinal serious adverse events in a commercially-insured population. *Pharmacoepidemiol Drug Saf.* 2010;19(6):596-603.
23. Andrade SE, Harrold LR, Tjia J, et al. A systematic review of validated methods for identifying cerebrovascular accident or transient ischemic attack using administrative data. *Pharmacoepidemiol Drug Saf.* 2012;21 Suppl 1:100-128.
24. Tirschwell DL, Longstreth WT. Validating administrative data in stroke research. *Stroke.* 2002;33(10):2465-2470.
25. Roumie CL, Mitchel E, Gideon PS, Varas-Lorenzo C, Castellsague J, Griffin MR. Validation of ICD-9 codes with a high positive predictive value for incident strokes resulting in hospitalization using Medicaid health data. *Pharmacoepidemiol Drug Saf.* 2008;17(1):20-26.
26. da Graca B, Filardo G, Nicewander D. Consequences for healthcare quality and research of the exclusion of records from the death master file. *Circulation: Cardiovascular Quality and Outcomes.* 2013;6(1):124-128.
27. Austin PC. Optimal caliper widths for propensity-score matching when estimating differences in means and differences in proportions in observational studies. *Pharm Stat.* 2011;10(2):150-161.
28. Austin PC. Balance diagnostics for comparing the distribution of baseline covariates between treatment groups in propensity-score matched samples. *Stat Med.* 2009;28(25):3083-3107.
29. Gayat E, Resche-Rigon M, Mary JY, Porcher R. Propensity score applied to survival data analysis through proportional hazards models: a Monte Carlo study. *Pharm Stat.* 2012;11(3):222-229.
30. Grambsch PM, Therneau TM. Proportional hazards test and diagnostics base don weighted residuals. Biometrika. 1994;81(3):515-526.
31. Fine JP, Gray RJ. A proportional hazards model for the subdistribution of a competing risk. *Journal of the American statistical association.* 1999;94(446):496-509.
32. Prasad V, Jena AB. Prespecified falsification end points: can they validate true observational associations? *JAMA.* 2013;309(3):241-242.
33. Dusetzina SB, Brookhart MA, Maciejewski ML. Control Outcomes and Exposures for Improving Internal Validity of Nonrandomized Studies. *Health Serv Res.* 2015;50(5):1432-1451.

© 2021 Wallach JD et al. *JAMA Network Open.*

34. Ioannidis JP. Are mortality differences detected by administrative data reliable and actionable? *Jama.* 2013;309(13):1410-1411.
35. Lipsitch M, Tchetgen Tchetgen E, Cohen T. Negative controls: a tool for detecting confounding and bias in observational studies. *Epidemiology.* 2010;21(3):383-388.
36. Austin PC, Stuart EA. Moving towards best practice when using inverse probability of treatment weighting (IPTW) using the propensity score to estimate causal treatment effects in observational studies. *Stat Med.* 2015;34(28):3661-3679.
37. Wallach JD, Ciani O, Pease AM, et al. Comparison of treatment effect sizes from pivotal and postapproval trials of novel therapeutics approved by the FDA based on surrogate markers of disease: a meta-epidemiological study. *BMC Med.* 2018;16(1):45.

© 2021 Wallach JD et al. *JAMA Network Open.*

eFigure. Initial Cohort for PRONOUNCE

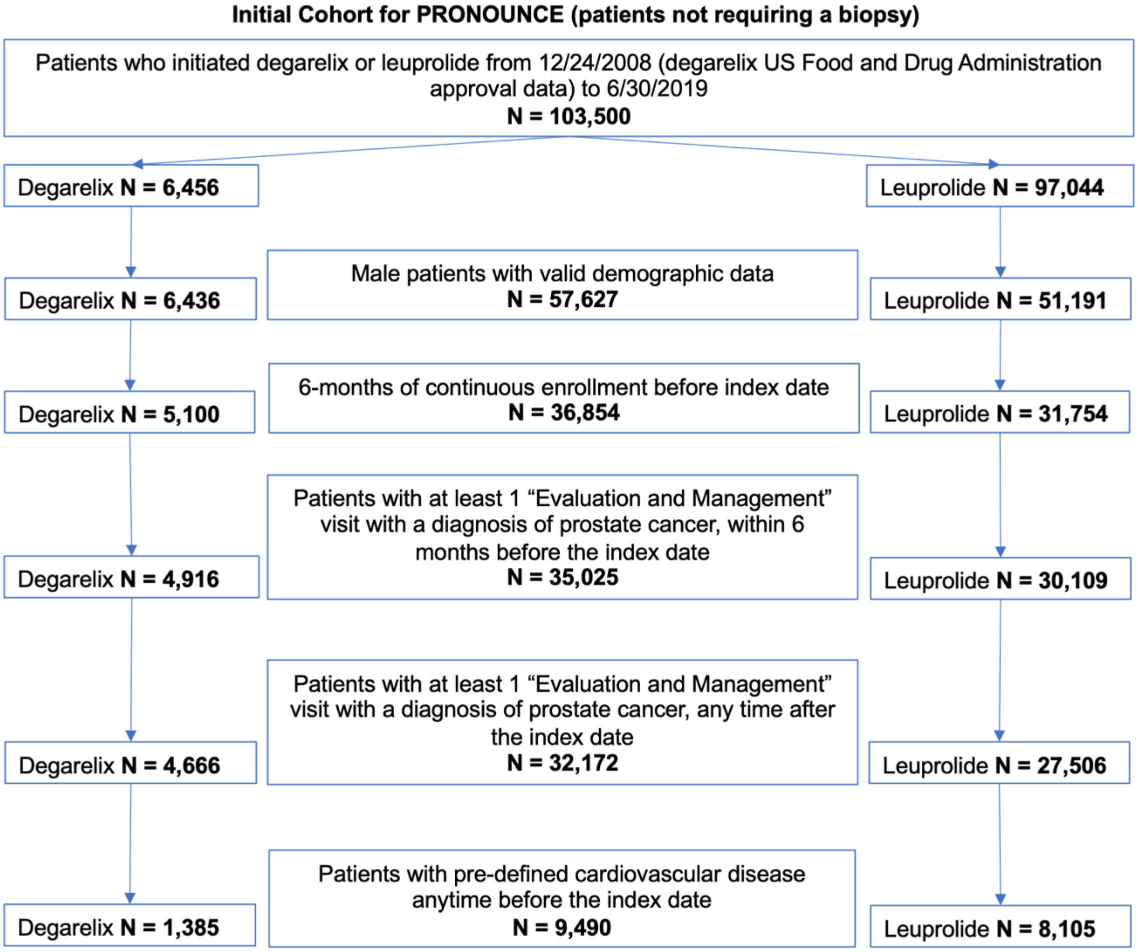

© 2021 Wallach JD et al. *JAMA Network Open.*

eTable 1. Summary of Inclusion and Exclusion Criteria for PRONOUNCE Trial, Overall Cohort Population and Postcohort Development

**A. Summary of Inclusion/Exclusion Criteria for PRONOUNCE Trial (No biopsy required): overall cohort population (n=32172)**				
**PRONOUNCE Eligibility Criteria**	**Operational Definition in OLDW**	**Overall Cohort (N=32172)**	**Degarelix (Firmagon) (N =4666)**	**Leuprolide (Lupron Depot) (N =27506)**
Total % of patients eligible for PRONOUNCE		7800 (24.2%)	1120 (24.0%)	6680 (24.3%)
Total % of patients ineligible for PRONOUNCE		24372 (75.8)	3546 (76.0)	20826 (75.7)
Patients who did NOT meet the inclusion criteria below (%)				
Histologically confirmed adenocarcinoma of the prostate Tumor, node, metastasis staging available prior to treatment start (bone scan and/or CT scan and/or MRI) <12 weeks prior to study start. If no radiographic image is available at the time of screening, a bone scan should be performed	NA. Already defined in the initial cohort as: *Patients must have at least one "Evaluation and Management" visit with a diagnosis of prostate cancer within 6 months before index data, at least one "Evaluation and Management" visit with a diagnosis of prostate cancer at any time after the index date*	-	-	-
Indication to initiate androgen deprivation therapy (ADT)	NA. 'Index date' is the first fill of degarelix or leuprolide*	-	-	-
Predefined cardiovascular disease inclusion criteria				

© 2021 Wallach JD et al. *JAMA Network Open.*

Pre-existing ASCVD (confirmed diagnosis, documented) according to at least one of the following criteria:<br>Prior myocardial infarction >=30 days before randomization; prior revascularization procedure >=30 days before randomization);<br>Coronary artery: stent placement/balloon angioplasty or coronary artery bypass graft surgery; Coronary artery: stent placement/balloon angioplasty or endarterectomy surgery;<br>Iliac, femoral, popliteal arteries: stent placement/balloon angioplasty or vascular bypass surgery<br>At least one vascular stenosis >=50% at any time point before randomization by angiogram or CT angiogram<br>Coronary artery<br>Carotid artery<br>Iliac femoral, or popliteal arteries<br>Carotid ultrasound results that documented a vascular stenosis >-50% at any time point before randomization<br>Ankle-brachial pressure index <0.9 at any time point before randomization.	NA. Already defined in the initial cohort as:<br><br>Diagnosis and procedure codes at any position for the following cardiovascular events, >= 30 days before the index date: Myocardial infarction (MI), percutaneous coronary intervention (PCI), coronary artery bypass grafting(CABG), Peripheral artery disease (PAD), peripheral artery revascularization, carotid revascularization<br><br>*We do not have angiogram, CT angiogram, or ankle-brachial pressure data.*	22682 (70.5%)	3281 (70.3%)	19401 (70.5%)
<u>Patients who met one of the following exclusion criteria (%)</u>				

© 2021 Wallach JD et al. *JAMA Network Open.*

Previous or current hormonal management of prostate cancer<br><br>- Surgical castration<br>- Any hormonal manipulation<br><br>Except prior neoadjuvant/adjuvant hormonal therapy, in this case treatment must be terminated >12 months prior to study start.	A prescription fill of ADT medications within 6 months before the index date<br>*(ADT medications include: Abiraterone acetate, apalutamide, bicalutamide, buserelin, degarelix, diethylstilbestrol, enzalutamide, flutamide, goserelin acetate, histrelin acetate, leuprolide acetate, nilutamide, triptorelin pamoate)*<br>*OR*<br>Procedure codes for bilateral orchiectomy within 6 months before the index date.<br><br>**For the leuprolide cohort, we will allow up to one month of bicalutamide prior to leuprolide initiation*	3213 (10.0%)	470 (10.1%)	2,743 (10.0%)
Main cardiovascular exclusion criteria				
Uncontrolled type 1 or type 2 diabetes mellitus (defined as HbA1c >	NA. Restricting patients with laboratory data	-	-	-

© 2021 Wallach JD et al. *JAMA Network Open.*

10%) at time of randomization	would limit the sample.			
Uncontrolled hypertension (SBP >180 mmHg or DBP >110 mmHg) at time of randomization	NA. Restricting patients with laboratory data would limit the sample.	-	-	-
A history of congenital long QT syndrome or risk factors for torsade de pointes ventricular arrhythmias (e.g., heart failure, hypokalemia, concomitant medication known to cause QT prolongation)	NA. This exclusion criteria only applies to patients at risk for ventricular arrhythmias.	-	-	-
Within 30 days prior to randomization<br>- Myocardial infarction<br>- Stroke (hemorrhagic)<br>- Coronary, carotid, or peripheral artery revascularization	Primary diagnosis of myocardial infarction and stroke (emergency department or inpatient visits) within 30 days before index date Percutaneous coronary intervention (PCI), coronary artery bypass grafting (CABG), peripheral artery revascularization (limb events), carotid revascularization within 30 days before index date	822 (2.6%)	140 (3.0%)	682 (2.5%)
Planned or scheduled cardiac surgery or PCI procedure that is known at the time of randomization	NA. We cannot determine planned or scheduled PCI procedures.	-	-	-

© 2021 Wallach JD et al. *JAMA Network Open.*

Ankle-brachial pressure index <0.9 at any point before randomization	NA. Restricting patients with laboratory data would limit the sample.	-	-	-

B. Summary of Inclusion/Exclusion Criteria for PRONOUNCE Trial (No biopsy required): post-cohort development (n=9490; eFigure 1)				
**PRONOUNCE Eligibility Criteria**	**Operational Definition in OLDW**	**Overall Cohort (N=9490)**	**Degarelix (Firmagon) (N =1385)**	**Leuprolide (Lupron Depot) (N =8105)**
Total % of patients eligible for PRONOUNCE		7800 (82.2%)	1120 (80.9%)	6680 (82.4%)
Total % of patients ineligible for PRONOUNCE		1690 (17.8%)	265 (19.1%)	1425 (17.6%)
Patients who did NOT meet the inclusion criteria below (%)				
Histologically confirmed adenocarcinoma of the prostate Tumor, node, metastasis staging available prior to treatment start (bone scan and/or CT scan and/or MRI) <12 weeks prior to study start. If no radiographic image is available at the time of screening, a bone scan should be performed	NA. Already defined in the initial cohort as:<br><br>*Patients must have at least one “Evaluation and Management” visit with a diagnosis of prostate cancer within 6 months before index data, at least one “Evaluation and Management” visit with a diagnosis of prostate cancer at any time after the index date*	-	-	-
Indication to initiate androgen deprivation therapy (ADT)	NA. ‘Index date’ is the first fill of degarelix or leuprolide*	-	-	-

© 2021 Wallach JD et al. *JAMA Network Open.*

Predefined cardiovascular disease inclusion criteria				
Pre-existing ASCVD (confirmed diagnosis, documented) according to at least one of the following criteria:<br>Prior myocardial infarction >=30 days before randomization; prior revascularization procedure >=30 days before randomization);<br>Coronary artery: stent placement/balloon angioplasty or coronary artery bypass graft surgery; Coronary artery: stent placement/balloon angioplasty or endarterectomy surgery;<br>Iliac, femoral, popliteal arteries: stent placement/balloon angioplasty or vascular bypass surgery<br>At least one vascular stenosis >=50% at any time point before randomization by angiogram or CT angiogram<br>Coronary artery<br>Carotid artery<br>Iliac femoral, or popliteal arteries<br>Carotid ultrasound results that documented a vascular stenosis >-50% at any time point before randomization<br>Ankle-brachial pressure index <0.9 at any time point before randomization.	NA. Already defined in the initial cohort as:<br><br>Diagnosis and procedure codes at any position for the following cardiovascular events, >= 30 days before the index date: Myocardial infarction (MI), percutaneous coronary intervention (PCI), coronary artery bypass grafting(CABG), Peripheral artery disease (PAD), peripheral artery revascularization, carotid revascularization<br><br>*We do not have angiogram, CT angiogram, or ankle-brachial pressure data.*	-	-	-

© 2021 Wallach JD et al. *JAMA Network Open.*

Patients who met one of the following exclusion criteria (%)				
Previous or current hormonal management of prostate cancer<br><br>- Surgical castration<br>- Any hormonal manipulation<br><br>Except prior neoadjuvant/adjuvant hormonal therapy, in this case treatment must be terminated >12 months prior to study start.	A prescription fill of ADT medications within 6 months before the index date<br>*(ADT medications include: Abiraterone acetate, apalutamide, bicalutamide, buserelin, degarelix, diethylstilbestrol, enzalutamide, flutamide, goserelin acetate, histrelin acetate, leuprolide acetate, nilutamide, triptorelin pamoate)*<br>*OR*<br>Procedure codes for bilateral orchiectomy within 6 months before the index date.<br><br>**For the leuprolide cohort, we will allow up to one month of bicalutamide prior to leuprolide initiation*	989 (10.4%)	150 (10.8%)	839 (10.4%)
Main cardiovascular exclusion criteria				

© 2021 Wallach JD et al. *JAMA Network Open.*

Uncontrolled type 1 or type 2 diabetes mellitus (defined as HbA1c > 10%) at time of randomization	NA. Restricting patients with laboratory data would limit the sample.	-	-	-
Uncontrolled hypertension (SBP >180 mmHg or DBP >110 mmHg) at time of randomization	NA. Restricting patients with laboratory data would limit the sample.	-	-	-
A history of congenital long QT syndrome or risk factors for torsade de pointes ventricular arrhythmias (e.g., heart failure, hypokalemia, concomitant medication known to cause QT prolongation)	NA. This exclusion criteria only applies to patients at risk for ventricular arrhythmias.	-	-	-
Within 30 days prior to randomization<br>- Myocardial infarction<br>- Stroke (hemorrhagic)<br>- Coronary, carotid, or peripheral artery revascularization	Primary diagnosis of myocardial infarction and stroke (emergency department or inpatient visits) within 30 days before index date Percutaneous coronary intervention (PCI), coronary artery bypass grafting (CABG), peripheral artery revascularization (limb events), carotid revascularization within 30 days before index date	789 (8.3%)	133 (9.6%)	656 (8.1%)
Planned or scheduled cardiac surgery or PCI procedure that is known at the time of randomization	NA. We cannot determine planned or	-	-	-

© 2021 Wallach JD et al. *JAMA Network Open.*

	scheduled PCI procedures.			
Ankle-brachial pressure index <0.9 at any point before randomization	NA. Restricting patients with laboratory data would limit the sample.	-	-	-

© 2021 Wallach JD et al. *JAMA Network Open.*

eTable 2. Use of Hormonal Management of Prostate Cancer and First-Generation Antiandrogen Agents Within 6 Months After Index Date and Baseline Characteristics Among Patients With and Without Prostate-Specific Antigen Levels

**A. Concurrent use of hormonal management of prostate cancer and first-generation antiandrogen agents within 6 month after index date**			
**Concurrent use of hormonal management of prostate cancer (after index date)**	Degarelix (N=1113)	Leuprolide (N=1113)	Total (N=2226)
Abiraterone acetate	43 (3.9%)	26 (2.3%)	69 (3.1%)
Apalutamide	0	0	0
Buserelin	0	0	0
Cyproterone	0	0	0
Enzalutamide	* (<1%)	* (<1%)	14 (0.6%)
Goserelin acetate	0	0	0
Histrelin acetate	* (<1%)	* (<1%)	* (<1%)
Triptorelin pamoate	56 (5.0%)	* (<1%)	* (2.6%)
Bilateral orchiectomy	*(<1%)	* (<1%)	* (<1%)
**Concurrent use of first-generation antiandrogen agent (after index date)**			
Flutamide	0	* (<1%)	* (<1%)
Bicalutamide	54 (4.9%))	286 (25.7%)	340 (15.3%)
Nilutamide	0	0	0
* Cell suppression based on OptumLabs Cell Size Suppression rules. N<11 are masked to protect patient confidentiality			

**B. Baseline characteristics among patients with and without prostate-specific antigen (PSA) levels lab data**						
	**With PSA**			**Without PSA**		
	**Degarelix (N=395)**	**Leuprolide (N=2246)**	**Total (N=2641)**	**Degarelix (N=725)**	**Leuprolide (N=4434)**	**Total (N=5159)**
**Age**						
Mean (SD)	74.2 (7.7)	74.0 (7.5)	74.0 (7.5)	75.5 (7.5)	74.4 (7.3)	74.6 (7.3)
Median	74.0	74.0	74.0	76.0	75.0	75.0
Q1, Q3	69.0, 81.0	69.0, 80.0	69.0, 80.0	70.0, 82.0	70.0, 80.0	70.0, 80.0
**Age group**						
<=54	<11*	>19*	>23*	<11*	>21*	>24*
55-64	>40*	>216*	>261*	>68*	>431*	>498*
65-74	148 (37.5 %)	879 (39.1 %)	1027 (38.9 %)	240 (33.1 %)	1539 (34.7 %)	1779 (34.5 %)
75+	197 (49.9 %)	1120 (49.9 %)	1317 (49.9 %)	416 (57.4 %)	2425 (54.7 %)	2841 (55.1 %)

© 2021 Wallach JD et al. *JAMA Network Open.*

**Race/Ethnicity**						
Asian	13 (3.3%)	62 (2.8%)	75 (2.8%)	<11*	>75*	>89*
Black	88 (22.3%)	382 (17.0%)	470 (17.8%)	>120*	>798*	>919*
Hispanic	20 (5.1%)	314 (14.0%)	334 (12.6%)	35 (4.8%)	294 (6.6%)	329 (6.4%)
White	258 (65.3%)	1407 (62.6%)	1665 (63.0%)	525 (72.4%)	3068 (69.2%)	3593 (69.6%)
Unknown	16 (4.1%)	81 (3.6%)	97 (3.7%)	33 (4.6%)	194 (4.4%)	227 (4.4%)
**Geographic Region**						
Midwest	45 (11.4%)	257 (11.4%)	302 (11.4%)	256 (35.3%)	1573 (35.5%)	1829 (35.5%)
Northeast	100 (25.3%)	373 (16.6%)	473 (17.9%)	117 (16.1%)	690 (15.6%)	807 (15.6%)
South	218 (55.2%)	1396 (62.2%)	1614 (61.1%)	286 (39.4%)	1764 (39.8%)	2050 (39.7%)
West	32 (8.1%)	>207*	>239	>54*	>391*	>455
Unknown	0 (0.0%)	<11*	<11*	<11*	<11*	<11*
**Serum PSA level**						
N	395	2246	2641	0	0	0
Mean (SD)	72.9 (271.0)	48.9 (290.7)	52.5 (287.9)	NA	NA	NA
Median	11.6	9.2	9.4	NA	NA	NA
Q1, Q3	6.2, 32.4	5.1, 19.8	5.2, 21.1	NA	NA	NA
**Estimated Glomerular Filtration Rate (eGFR)**						
N	293	1705	1998	140	648	788
Mean (SD)	68.1 (20.3)	67.6 (20.0)	67.7 (20.1)	63.2 (20.6)	64.7 (21.3)	64.4 (21.2)
Median	70.4	69.4	69.5	63.0	66.2	65.7
Q1, Q3	54.9, 82.5	53.7, 83.0	53.9, 82.9	48.5, 80.5	51.0, 81.1	50.8, 81.1
**Prior prostate biopsy (within 6 months of index date)**	266 (67.3%)	1306 (58.1%)	1572 (59.5%)	416 (57.4%)	2329 (52.5%)	2745 (53.2%)

© 2021 Wallach JD et al. *JAMA Network Open.*

**Baseline comorbidities**						
Coronary artery disease	199 (50.4 %)	1061 (47.2 %)	1260 (47.7 %)	394 (54.3 %)	2363 (53.3 %)	2757 (53.4 %)
Chronic kidney disease	51 (12.9%)	314 (14.0 %)	365 (13.8 %)	116 (16.0 %)	563 (12.7 %)	679 (13.2 %)
Congestive heart failure	63 (15.9%)	299 (13.3 %)	362 (13.7 %)	128 (17.7 %)	742 (16.7 %)	870 (16.9 %)
Cerebrovascular disease	51 (12.9%)	292 (13.0 %)	343 (13.0 %)	108 (14.9 %)	593 (13.4 %)	701 (13.6 %)
Peripheral vascular disease	101 (25.6 %)	697 (31.0 %)	798 (30.2 %)	225 (31.0 %)	1304 (29.4 %)	1529 (29.6 %)
Obesity	25 (6.3%)	176 (7.8%)	201 (7.6%)	46 (6.3%)	275 (6.2%)	321 (6.2%)
Atrial Fibrillation	59 (14.9%)	359 (16.0 %)	418 (15.8 %)	132 (18.2 %)	745 (16.8 %)	877 (17.0 %)
Sleep apnea	36 (9.1%)	214 (9.5%)	250 (9.5%)	71 (9.8%)	468 (10.6 %)	539 (10.4 %)
Hypertension	320 (81.0 %)	1793 (79.8 %)	2113 (80.0 %)	562 (77.5 %)	3497 (78.9 %)	4059 (78.7 %)
MI	41 (10.4%)	211 (9.4%)	252 (9.5%)	78 (10.8%)	485 (10.9 %)	563 (10.9 %)
Stroke	22 (5.6%)	116 (5.2%)	138 (5.2%)	38 (5.2%)	259 (5.8%)	297 (5.8%)
PCI	28 (7.1%)	164 (7.3%)	192 (7.3%)	53 (7.3%)	330 (7.4%)	383 (7.4%)
CABG	31 (7.8%)	213 (9.5%)	244 (9.2%)	66 (9.1%)	421 (9.5%)	487 (9.4%)
PAD	71 (18.0%)	485 (21.6 %)	556 (21.1 %)	161 (22.2 %)	854 (19.3 %)	1015 (19.7 %)
Dementia	21 (5.3%)	71 (3.2%)	92 (3.5%)	36 (5.0%)	200 (4.5%)	236 (4.6%)
COPD	86 (21.8%)	442 (19.7 %)	528 (20.0 %)	158 (21.8 %)	898 (20.3 %)	1056 (20.5 %)
Peptic ulcer disease	<11*	>20*	>21*	<11*	>42*	>49*
Mild liver disease	35 (8.9%)	196 (8.7%)	231 (8.7%)	43 (5.9%)	328 (7.4%)	371 (7.2%)
Diabetes without chronic complication	155 (39.2 %)	845 (37.6 %)	1000 (37.9 %)	259 (35.7 %)	1560 (35.2 %)	1819 (35.3 %)
Diabetes with chronic complication	77 (19.5%)	364 (16.2 %)	441 (16.7 %)	105 (14.5 %)	647 (14.6 %)	752 (14.6 %)
Metastatic solid tumor	81 (20.5%)	333 (14.8 %)	414 (15.7 %)	170 (23.4 %)	764 (17.2 %)	934 (18.1 %)

© 2021 Wallach JD et al. *JAMA Network Open.*

Rheumatic disease	<11*	>44*	>49*	15 (2.1%)	100 (2.3%)	115 (2.2%)
**Charlson comorbidity score**						
Mean (SD)	5.4 (3.2)	5.0 (2.9)	5.1 (2.9)	5.6 (3.3)	5.1 (3.1)	5.2 (3.1)
Median	4.0	4.0	4.0	5.0	4.0	4.0
Q1, Q3	3.0, 8.0	3.0, 7.0	3.0, 7.0	3.0, 8.0	3.0, 7.0	3.0, 7.0
**Prior radiotherapy (within 6 months before index date)**	<11*	>71*	>77*	13 (1.8%)	132 (3.0%)	145 (2.8%)
**Baseline medications (within 6 months before index date)**						
Statin	259 (65.6 %)	1437 (64.0 %)	1696 (64.2 %)	458 (63.2 %)	2827 (63.8 %)	3285 (63.7 %)
Non-statin lipid lower medications	59 (14.9%)	314 (14.0 %)	373 (14.1 %)	111 (15.3 %)	662 (14.9 %)	773 (15.0 %)
ACEi	150 (38.0 %)	815 (36.3 %)	965 (36.5 %)	246 (33.9 %)	1643 (37.1 %)	1889 (36.6 %)
ARB	90 (22.8%)	456 (20.3 %)	546 (20.7 %)	125 (17.2 %)	787 (17.7 %)	912 (17.7 %)
ACEi/ARB	230 (58.2 %)	1248 (55.6 %)	1478 (56.0 %)	363 (50.1 %)	2372 (53.5 %)	2735 (53.0 %)
Sacubitril / Valsartan	<11*	<11*	<11*	<11*	<11*	>11*
Warfarin	28 (7.1%)	176 (7.8%)	204 (7.7%)	74 (10.2%)	472 (10.6 %)	546 (10.6 %)
DOAC	27 (6.8%)	113 (5.0%)	140 (5.3%)	43 (5.9%)	185 (4.2%)	228 (4.4%)
Beta-blockers	201 (50.9 %)	1044 (46.5 %)	1245 (47.1 %)	380 (52.4 %)	2167 (48.9 %)	2547 (49.4 %)
Loop diuretics	52 (13.2%)	301 (13.4 %)	353 (13.4 %)	122 (16.8 %)	741 (16.7 %)	863 (16.7 %)
Aldosterone antagonist	16 (4.1%)	70 (3.1%)	86 (3.3%)	30 (4.1%)	160 (3.6%)	190 (3.7%)
Digoxin	<11*	>65*	>74*	19 (2.6%)	139 (3.1%)	158 (3.1%)

© 2021 Wallach JD et al. *JAMA Network Open.*

Calcium channel blocker	118 (29.9%)	596 (26.5%)	714 (27.0%)	176 (24.3%)	1070 (24.1%)	1246 (24.2%)
Antiplatelet	77 (19.5%)	407 (18.1%)	484 (18.3%)	137 (18.9%)	812 (18.3%)	949 (18.4%)
**Number of hospitalizations**						
0	335 (84.8%)	1928 (85.8%)	2263 (85.7%)	587 (81.0%)	3601 (81.2%)	4188 (81.2%)
1	48 (12.2%)	269 (12.0%)	317 (12.0%)	105 (14.5%)	646 (14.6%)	751 (14.6%)
2+	12 (3.0%)	49 (2.2%)	61 (2.3%)	33 (4.6%)	187 (4.2%)	220 (4.3%)
**Number of ER visit**						
0	310 (78.5%)	1791 (79.7%)	2101 (79.6%)	527 (72.7%)	3360 (75.8%)	3887 (75.3%)
1	53 (13.4%)	294 (13.1%)	347 (13.1%)	111 (15.3%)	641 (14.5%)	752 (14.6%)
2+	32 (8.1%)	161 (7.2%)	193 (7.3%)	87 (12.0%)	433 (9.8%)	520 (10.1%)
**Year of Cohort Entry**						
2008	0 (0.0%)	0 (0.0%)	0 (0.0%)	0 (0.0%)	0 (0.0%)	0 (0.0%)
2009	0 (0.0%)	92 (4.1%)	92 (3.5%)	0 (0.0%)	285 (6.4%)	285 (5.5%)
2010	<11*	>89*	>89*	25 (3.4%)	345 (7.8%)	370 (7.2%)
2011	<11*	>90*	>90*	50 (6.9%)	390 (8.8%)	440 (8.5%)
2012	18 (4.6%)	124 (5.5%)	142 (5.4%)	55 (7.6%)	358 (8.1%)	413 (8.0%)
2013	29 (7.3%)	163 (7.3%)	192 (7.3%)	60 (8.3%)	310 (7.0%)	370 (7.2%)
2014	39 (9.9%)	169 (7.5%)	208 (7.9%)	56 (7.7%)	312 (7.0%)	368 (7.1%)
2015	39 (9.9%)	217 (9.7%)	256 (9.7%)	65 (9.0%)	362 (8.2%)	427 (8.3%)
2016	62 (15.7%)	288 (12.8%)	350 (13.3%)	88 (12.1%)	454 (10.2%)	542 (10.5%)
2017	70 (17.7%)	365 (16.3%)	435 (16.5%)	128 (17.7%)	662 (14.9%)	790 (15.3%)
2018	86 (21.8%)	448 (19.9%)	534 (20.2%)	139 (19.2%)	708 (16.0%)	847 (16.4%)
2019	37 (9.4%)	186 (8.3%)	223 (8.4%)	59 (8.1%)	246 (5.5%)	305 (5.9%)
**State**						
AL	14 (3.5%)	56 (2.5%)	70 (2.7%)	20 (2.8%)	114 (2.6%)	134 (2.6%)

AZ	12 (3.0%)	68 (3.0%)	80 (3.0%)	<11*	>30*	>30*
CA	<11*	>30*	>30*	<11*	>70*	>70*
CT	<11*	>30*	>30*	19 (2.6%)	107 (2.4%)	126 (2.4%)
FL	84 (21.3%)	580 (25.8 %)	664 (25.1 %)	80 (11.0%)	476 (10.7 %)	556 (10.8 %)
GA	17 (4.3%)	168 (7.5%)	185 (7.0%)	37 (5.1%)	327 (7.4%)	364 (7.1%)
IL	13 (3.3%)	54 (2.4%)	67 (2.5%)	49 (6.8%)	264 (6.0%)	313 (6.1%)
IN	12 (3.0%)	23 (1.0%)	35 (1.3%)	27 (3.7%)	154 (3.5%)	181 (3.5%)
MA	<11*	<11*	<11*	<11*	>80*	>80*
MN	0 (0.0%)	0 (0.0%)	0 (0.0%)	12 (1.7%)	157 (3.5%)	169 (3.3%)
MO	<11%	>55*	>55*	41 (5.7%)	213 (4.8%)	254 (4.9%)
NC	53 (13.4%)	120 (5.3%)	173 (6.6%)	72 (9.9%)	253 (5.7%)	325 (6.3%)
NJ	47 (11.9%)	119 (5.3%)	166 (6.3%)	28 (3.9%)	115 (2.6%)	143 (2.8%)
NY	40 (10.1%)	166 (7.4%)	206 (7.8%)	49 (6.8%)	236 (5.3%)	285 (5.5%)
OH	<11%	>55*	>55*	28 (3.9%)	269 (6.1%)	297 (5.8%)
RI	<11%	>30*	>30*	12 (1.7%)	80 (1.8%)	92 (1.8%)
SC	13 (3.3%)	39 (1.7%)	52 (2.0%)	16 (2.2%)	70 (1.6%)	86 (1.7%)
TN	<11%	>25*	37 (1.4%)	12 (1.7%)	100 (2.3%)	112 (2.2%)
TX	11 (2.8%)	274	285 (10.8 %)	17 (2.3%)	259 (5.8%)	276 (5.3%)
UT	<11%	>40*	>40*	12 (1.7%)	100 (2.3%)	112 (2.2%)
VA	<11%	>30*	>30*	<11%	>40*	>40*
WI	<11%	>35*	>35*	70 (9.7%)	330 (7.4%)	400 (7.8%)
OTHER	34 (8.6%)	186 (8.3%)	220 (8.3%)	103 (14.2 %)	558 (12.6 %)	661 (12.8 %)

ACEi, angiotensin-converting-enzyme inhibitor; ARB, angiotensin II receptor blocker; CABG, coronary artery bypass grafting; COPD, chronic obstructive pulmonary disease; DOAC, direct-acting oral anticoagulant; IQR, interquartile range; MI = myocardial infarction; PAD, peripheral artery disease; PCI, percutaneous coronary intervention; PSA prostate-specific antigen

[a] Patients with PSA and patients without PSA were matched separately

[b] eGFR was used for the subgroup analyses and not for matching purposes

* Cell suppression based on OptumLabs Cell Size Suppression rules. N<11 are masked to protect patient confidentiality

© 2021 Wallach JD et al. *JAMA Network Open.*

eTable 3. Subgroup Analyses for the Primary Intention-to-Treat Propensity Score Matched Analyses										
**MACE**	**Degarelix**				**Leuprolide**				**Effect Estimate**	
**Subgroup**	No. of Patients	No. of Events	Person-Years	Event Rate	No. of Patients	No. of Events	Person-Years	Event Rate	HR (95% CI)	P-value for interaction
**All Patients**	1113	88	864.65	10.18	1113	73	849.15	8.60	1.18 (0.86, 1.61)	
**Age Category**										
<=64	117	NA*	NA*	NA*	107	NA*	NA*	NA*	0.40 (0.10, 1.56)	0.26
65-74	385	20	302.76	6.61	367	16	279.53	5.72	1.15 (0.60, 2.22)	
75+	611	65	465.21	13.97	639	51	488.90	10.43	1.34 (0.93, 1.93)	
**Race**										
White	778	61	602.93	10.12	779	51	593.62	8.59	1.17 (0.81, 1.70)	0.33
Black	207	19	163.34	11.63	208	14	158.68	8.82	1.30 (0.65, 2.60)	
Hispanic	55	NA*	NA*	NA*	51	NA*	NA*	NA*	0.29 (0.06, 1.46)	
Asian	24	NA*	NA*	NA*	27	NA*	NA*	NA*	NA	
**Diabetes**										
No	680	53	531.27	9.98	707	43	545.80	7.88	1.26 (0.84, 1.89)	0.58
Yes	433	35	333.38	10.50	406	30	303.35	9.89	1.05 (0.65, 1.72)	
**End-stage renal disease (ESRD)**										
No	1087	83	845.76	9.81	1086	70	829.98	8.43	1.16 (0.84, 1.59)	0.60
Yes	26	NA*	NA*	NA*	27	NA*	NA*	NA*	1.71 (0.41, 7.20)	

© 2021 Wallach JD et al. *JAMA Network Open.*

**Baseline eGFR**										
<45	67	NA*	NA*	NA*	71	NA*	NA*	NA*	1.00 (0.32, 3.10)	0.80
>=45	361	21	284.04	7.39	306	20	232.04	8.62	0.84 (0.46, 1.55)	

MACE, major adverse cardiovascular event; MI = myocardial infarction
Event rate was calculated as number of events per 100 person-years
* Cell suppression based on OptumLabs Cell Size Suppression rules. N<11 are masked to protect patient confidentiality

© 2021 Wallach JD et al. *JAMA Network Open.*

**eTable 4. Outcomes in Additional Subgroup Analyses**								
**Analysis**	**Number of Events**	**Person-years**	**Event rate**	**Number of Events**	**Person-years**	**Event rate**	**Hazard Ratio (95% CI)**	***P*-value**
**Patients with biopsy**	**Degarelix (N=727)**			**Leuprolide (N=718)**				
MACE	43	569.28	7.55	53	543.90	9.74	0.77 (0.52, 1.15)	0.21
Death	27	575.70	4.69	24	555.59	4.32	1.08 (0.62, 1.87)	0.79
MI	11	572.18	1.92	20	549.66	3.64	0.53 (0.25, 1.10)	0.09
Stroke	*	*	*	14	549.73	2.55	0.55 (0.23, 1.31)	0.18
Angina	*	*	*	*	*	*	0.95 (0.24, 3.81)	0.95

MACE, major adverse cardiovascular event; MI = myocardial infarction
Event rate was calculated as number of events per 100 person-years
* Cell suppression based on OptumLabs Cell Size Suppression rules. N<11 are masked to protect patient confidentiality

© 2021 Wallach JD et al. *JAMA Network Open.*

eTable 5. Falsification Analyses

	Leuprolide (N=1113)			Degarelix (N=1113)				
Outcomes	No. Events	Person-years	Rate per 100 person-years	No. Events	Person-years	Rate per 100 person-years	Hazard Ratio (95% CI)	p-value
COPD	22	852.14	2.58	24	865.45	2.77	1.07 (0.60, 1.90)	0.83
Appendicitis	0	*	*	*	*	*	*	*
Cholecystitis	*	*	*	*	*	*	0.74 (0.17, 3.31)	0.69

COPD, chronic obstructive pulmonary disease
*Cell suppression based on OptumLabs Cell Size Suppression rules. N<11 are masked to protect patient confidentiality

© 2021 Wallach JD et al. *JAMA Network Open.*
